# CircAST: Full-length Assembly and Quantification of Alternatively Spliced Isoforms in Circular RNAs

**DOI:** 10.1016/j.gpb.2019.03.004

**Published:** 2020-01-31

**Authors:** Jing Wu, Yan Li, Cheng Wang, Yiqiang Cui, Tianyi Xu, Chang Wang, Xiao Wang, Jiahao Sha, Bin Jiang, Kai Wang, Zhibin Hu, Xuejiang Guo, Xiaofeng Song

**Affiliations:** 1 Department of Biomedical Engineering, Nanjing University of Aeronautics and Astronautics, Nanjing 211106, China; 2 School of Biomedical Engineering and Informatics, Nanjing Medical University, Nanjing 211166, China; 3 State Key Laboratory of Reproductive Medicine, Nanjing Medical University, Nanjing 211166, China; 4 Center of Pathology and Clinical Laboratory, Sir Run Run Hospital, Nanjing Medical University, Nanjing 211166, China; 5 Center for Cellular and Molecular Therapeutic, Children’s Hospital of Philadelphia, Philadelphia, PA 19104, USA

**Keywords:** Circular RNA, Full-length reconstruction, Isoform quantification, Multiple splice graph model, Transcriptome

## Abstract

**Circular RNAs** (circRNAs), covalently closed continuous RNA loops, are generated from cognate linear RNAs through back splicing events, and alternative splicing events may generate different circRNA isoforms at the same locus. However, the challenges of reconstruction and quantification of alternatively spliced full-length circRNAs remain unresolved. On the basis of the internal structural characteristics of circRNAs, we developed CircAST, a tool to assemble alternatively spliced circRNA transcripts and estimate their expression by using multiple splice graphs. Simulation studies showed that CircAST correctly assembled the full sequences of circRNAs with a sensitivity of 85.63%–94.32% and a precision of 81.96%–87.55%. By assigning reads to specific isoforms, CircAST quantified the expression of circRNA isoforms with correlation coefficients of 0.85–0.99 between theoretical and estimated values. We evaluated CircAST on an in-house mouse testis RNA-seq dataset with RNase R treatment for enriching circRNAs and identified 380 circRNAs with full-length sequences different from those of their corresponding cognate linear RNAs. RT-PCR and Sanger sequencing analyses validated 32 out of 37 randomly selected isoforms, thus further indicating the good performance of CircAST, especially for isoforms with low abundance. We also applied CircAST to published experimental data and observed substantial diversity in circular transcripts across samples, thus suggesting that circRNA expression is highly regulated. CircAST can be accessed freely at https://github.com/xiaofengsong/CircAST.

## Introduction

Circular RNAs (circRNAs) are covalently closed continuous RNA loops generated through back splicing events [1], [2], [3], [4]. Recent studies have revealed the important roles of circRNAs in many biological processes, including normal brain function, myogenesis, and diseases such as glioma [5], [6], [7], [8], [9]. Accurate annotations based on the full-length sequences of circRNAs, such as miRNA sponge, protein binding sites, or protein-coding potential, are important for functional studies [6], [7], [8], [9], [10], [11], [12]. However, circRNAs may have multiple isoforms with different full-length sequences produced by alternative splicing (AS) events. For example, a recent study has shown that the sequence of the circRNA at the *FBXW7* locus is different from that of its cognate linear form and has revealed that the 185-aa protein encoded by *circFBXW7* inhibits cancer cell proliferation [9]. The lack of accurate full-length sequence information for circRNA poses significant challenges in functional studies. Furthermore, using the assembled circRNA isoforms with incorrect or incomplete sequences may result in erroneous conclusions in functional studies [9]. The full-length assembly and quantification of circRNA isoforms remain challenging problems, and novel computational methods to address these problems are urgently needed to facilitate research on circRNAs [13].

Previous efforts have reported only circRNA back splice sites that are different from those of the canonical linear transcripts, based on the notion that circRNA is a closed loop structure covalently linked by a downstream 5′ splice donor site and an upstream 3′ splice acceptor site. Therefore, tools find_circ [6], CIRCexplorer [14], CIRI [15], UROBORUS [16] and others [17], [18], [19], [20], [21], [22], [23] do not report the full-length sequences of circRNAs. Linear full-length assembly methods such as Cufflinks cannot use back-spliced reads, which are treated as unmapped reads and discarded in full-length assemblies [24]. CIRCexplorer2 uses Cufflinks to detect AS events on circRNAs from RNA-seq data, but it does not use all read information, and the use of a linear transcript model as a substitute could result in incorrect circRNA structures [25]. CIRI-AS uses long-read sequencing data to identify circRNA internal splicing structures [26]. CIRI-full can effectively reconstruct full-length circRNAs and perform isoform-level quantification from the transcriptome by combining reverse overlap and back splice junction (BSJ) features on the basis of long-read sequencing data [27]. CircTest estimates circRNA expression levels by using the ratio of circular BSJ read counts to the average total counts at exon boundaries [18]. Gu and colleagues have proposed Sailfish-cir to quantify circRNA expression by transforming a circular transcript into a pseudo-linear transcript for abundance estimation by using an existing linear model-based method, which cannot quantify the circRNAs with sequences different from those of the cognate linear forms [28]. Therefore, to address the problem of full-length assembly and quantification of circRNA, in this work, we developed CircAST. This tool uses multiple splice graphs as a circRNA model to reconstruct the full-length sequence and estimate expression levels by allocating reads to different isoforms with an expectation–maximization algorithm. CircAST can perform analysis downstream of existing back splice site detection software such as CIRCexplorer2, CIRI2, and UROBORUS [14], [15], [16]. Moreover, CircAST shows good performance in reconstructing and quantifying circular isoforms for diverse paired-end sequencing datasets.

## Method

### Circular transcript assembly using the extended minimum path cover algorithm

CircAST assembles circular transcripts from mapped fragments by using a multiple splice graph model. The assembler was designed to find a minimal set of circular transcripts that could explain all the fragments. In other words, each fragment should be contained in a certain circular transcript. CircAST first uses all spliced reads with one or more gaps corresponding to introns through alignment to the reference genome with Tophat2. These spliced reads, along with the information of exon boundaries provided in the gene annotation file, are used to construct splice graphs for all the back splicing events detected by upstream circRNA identification tools, such as UROBORUS, CIRCexplorer2, or CIRI2, in a gene locus. For each gene locus, multiple splice graphs are constructed if there is more than one back splicing event. Each splice graph is a directed acyclic graph (DAG) in which nodes indicate exons, and directed edges represent reads spanning two such nodes in the forward 5′ to 3′ order, thus indicating that they are consecutive exons in a circular transcript. Notably, the source and the sink of each DAG correspond to the two back-spliced exons of circRNAs in the genome. Each circular transcript should contain these two exons; thus, the path from source to sink can represent a possible full-length circular transcript (Figure 1).

**Figure 1:**
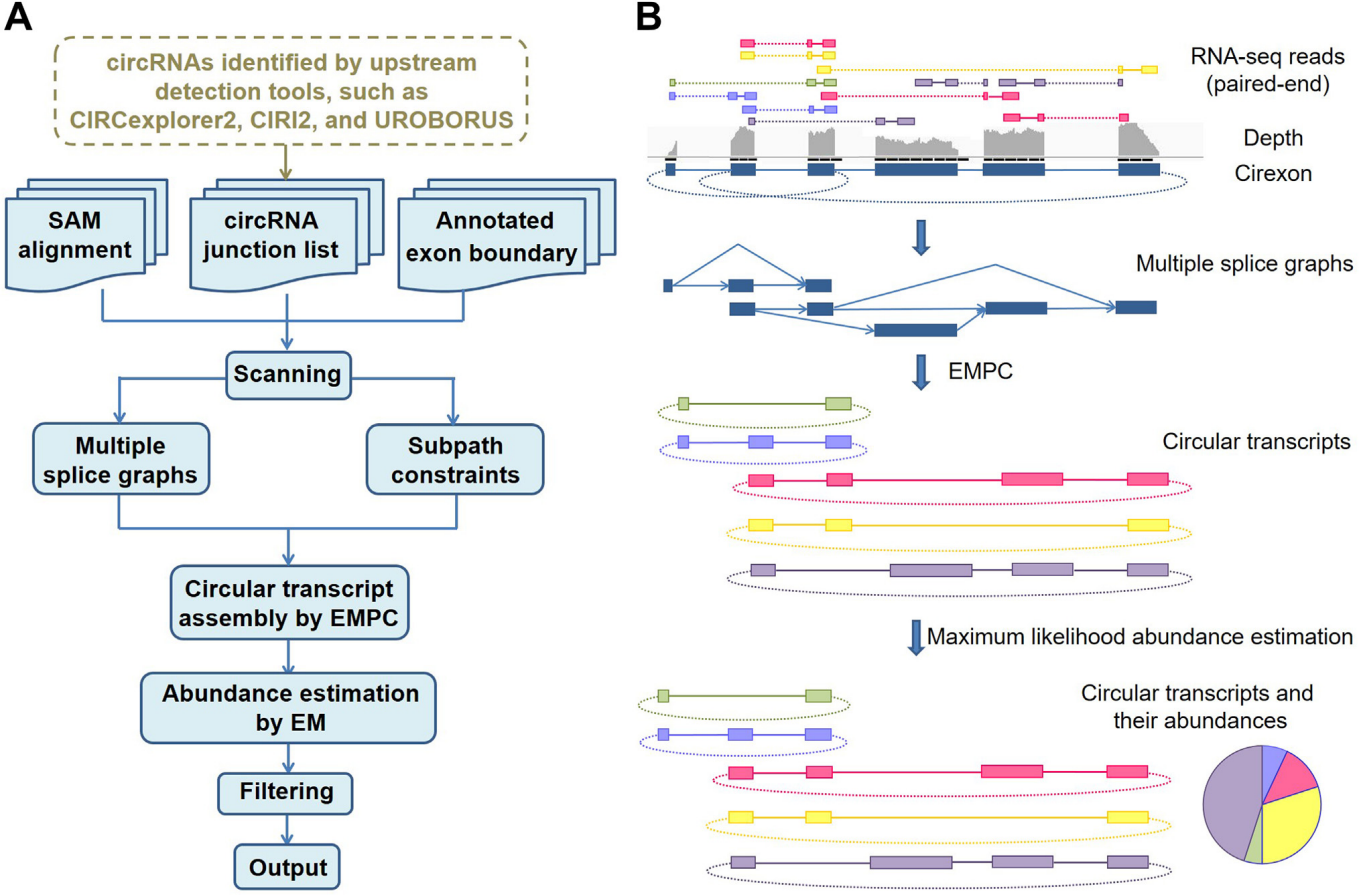
**Schematics of CircAST for circular transcript assembly and quantification** **A.** The flow diagram of CircAST. **B.** Visualization of the workflow of CircAST. CircAST begins with a set of paired-end RNA-seq reads that have been mapped to the genome. It then constructs multiple splice graphs with different BSJs in a gene locus and assembles the full-length sequences of circular transcripts with EMPC algorithm. CircAST estimates the abundance of each circular isoform assembled above by using an EM algorithm. Finally, all circular transcripts with full-length sequence assembly and abundance estimation are output in the results. SAM, sequence alignment/map; BSJ, back splice junction; EMPC, extended minimum path cover; EM, expectation maximization.

The linear transcript assembler usually constructs one graph at one gene locus on the basis of the given aligned reads, whereas CircAST builds multiple splice graphs corresponding to all the back splicing events in each gene locus. The number of splice graphs is equal to the number of back splicing events. Each splice graph contains only one single source and sink. The multiple splice graph model contains all the splicing events in the gene locus, including both forward- and back splicing events. If there are overlapping genomic regions among the back-spliced circRNAs, the fragments in these overlapping regions are shared among these splice graphs. Thus, the multiple splice graph model can solve the problem of multiple back splicing events in one gene.

After building the multiple splice graph model, CircAST solves the circular transcript assembly problem as an EMPC problem. A path cover of a directed graph G=(V,E) is a set of directed paths that cover all the vertexes v∈V. A minimum path cover (MPC) is the path cover with the minimal number of paths covering all the vertexes. The MPC problem is in general NP-complete, but it has been proven to be mathematically solvable in polynomial time on DAGs [29]. Therefore, the EMPC here extended the MPC problem to solve circular transcript assembly problems with multiple DAGs. The classic MPC problem is unsuitable for the circRNA assembly problem discussed above because it requires only that each node (exon) be covered at least once. However, in the actual splice graphs for circRNAs, each edge (spliced reads spanning two nodes) should also be covered at least once, and the source and sink of G should be contained in each path. Moreover, if long reads or paired-end reads are aligned to the nodes of G, there is a strong possibility that they may span more than two consecutive nodes (exons). These nodes constitute a subpath that should also be contained at least once in one of the final paths. Thus, EMPC was formulated as follows:

Given a series of DAGs $G_{i}=\left(V_{i},E_{i}\right)\left(i=1,2,\cdots n\right)$ in a multiple splice graph problem in one gene locus, each DAG $G_{i}$ has only one single source $S_{i}$ and sink $T_{i}$, and a subpath family $SP_{i}=\left\{sp_{i1},sp_{i2},\ldots ,sp_{\mathrm{im}}\right\}$. The objective is to find a minimum number k of directed paths $p_{1},p_{2},\ldots ,p_{k}$ subject to the following conditions: (a) every node in $V_{i}\left(G_{i}\right)$ shows up at least once in some $p_{j}$; (b) every edge in $E_{i}\left(G_{i}\right)$ shows up at least once in some $p_{j}$; (c) every subpath in $SP_{i}$ shows up at least once in some $p_{j}$; and (d) every path $p_{j}$ starts from the source $S_{i}$ and ends in the sink $T_{i}$ of some $G_{i}$.

Of note, in the construction of the DAGs and the search for subpaths, EMPC uses the fragments not only across the forward-spliced sites but also across the BSJ sites, the latter of which are missed in the linear transcript assembly algorithms (Figure S1A). Thus, EMPC makes use of all mapped fragments including circRNA-specific fragments. To find circRNAs with high confidence, CircAST filters the circular transcripts assembled by EMPC with extremely low expression abundance, at less than 1‰ of the most abundant isoform.

### Circular transcript abundance estimation

CircAST implements the expectation maximization (EM) algorithm to estimate the abundance of each assembled circRNA isoform. Given a set of paired-end RNA-seq data from an RNase R-treated sample, the statistical model is parameterized by the relative abundance $\left\{\theta_{t}\right\}$ of the circular transcript set T, and CircAST computes their maximum likelihood values. Because of the prevalence of AS, it is not known which transcript the fragment originates from, so that even given the read alignment to reference genome, the exact length of the fragment is unclear (Figure S1B). We denote the implied length of a fragment f by $l_{t}\left(f\right)$, assuming that it originates from transcript t. Unlike those of the linear transcripts, the lengths of circular transcripts $l_{t}\left(f\right)$ should be adjusted. When the circular transcript is much shorter than the fragment in length, the reads from the fragment termini could possibly be aligned to the same region in the circRNA (Figure S1C). In that case, the circular transcript length should be added to $l_{t}\left(f\right)$ manually. Considering a circular transcript t∈T with length l(t), the probability of selecting a fragment from t randomly is $\frac{1}{l(t)-2k+2}$, where k is the threshold of effective mapping and taken as 3 bp in our algorithm. Denoting the fragment distribution by F, which is assumed to be normal in CircAST, the likelihood function for our model is then given by:

$L\left(\theta |R\right)=\prod_{f\in R}\sum_{t\in T}\frac{\theta_{t}l\left(t\right)}{\sum_{\alpha \in T}\theta_{\alpha }l\left(\alpha \right)}\left(\frac{F[l_{t}\left(f\right)]}{l(t)-2k+2}\right)$

where the products are over all the fragment alignments R after all the likelihoods of transcripts T in the circular transcriptome are summarized. Many methods of linear transcript quantification, such as Cufflinks and Sailfish, use effective length normalization procedures on each transcript because of the existence of boundaries in mRNA [24], [30]. However, circRNA does not have boundaries and thus does not have ‘edge effects’ in deep sequencing. Therefore, l(t) should not be corrected for the quantification of circular transcripts. This model is non-negative and linear, and it can be statistically demonstrated that its log-likelihood function is concave and therefore has a unique maximum.

CircAST reports expression levels of circRNA transcripts in terms of fragments per kilobase million mapped reads (FPKM), transcripts per kilobase million mapped reads (TPM), and read counts in the full-length of circular transcripts. In previous studies, the reads of exon model per million mapped reads (RPM) metric has been used to evaluate the expression of circRNAs [14]. The number of BSJ reads is usually lower than that of all the reads mapped to the full-length of circRNA and is subject to large variations. RPM cannot quantify distinct isoforms from the same back splicing events. However, the metrics of FPKM, TPM, and read counts can remedy the gap and report the abundance of distinct isoforms from the same back splicing events in relative and absolute values separately.

### Definition of circRNA relation index

If circRNAs from multiple BSJ sites have overlapping genomic regions, reconstruction and abundance estimation of these circRNAs would increase in complexity. Therefore for each BSJ site, we define an relation index (RI) to evaluate the complexity, which is the overlapping degree of circRNAs derived from the BSJ site in the genome. Let R be a binary relation on the set J consisting of all the n BSJs in the dataset ($J=\{j_{1},j_{2},\cdots ,j_{n}\}$). For any $j_{s}$,$j_{t}\in J$, if the genomic regions between $j_{s}$ and $j_{t}$ are overlapping, we find that $j_{s}$ has a relation R with $j_{t}$, and denote this relationship as $j_{s}\text{R}j_{t}$. We define this binary relation with a transitivity property; that is, for the elements $j_{r}$,$j_{s}$,$j_{t}\in J$, if $j_{r}\text{R}j_{s}$ and $j_{s}\text{R}j_{t}$, then $j_{r}\text{R}j_{t}$. Clearly, R is an equivalence relation, and so it partitions the set J into k mutually exclusive and exhaustive equivalence classes $J_{1},J_{2},\cdots J_{k}$. The number of elements in each equivalence class, which is also called the cardinality of the class and is denoted as $\left|J_{1}\right|,\left|J_{2}\right|,\cdots \left|J_{k}\right|$, indicates the number of circRNAs with a mutual relationship in an overlapping genomic region. For each $j_{s}\in J\left(s=1,2,\cdots ,n\right)$, we define the RI as the cardinality of the equivalence class to which it belong; that is, $\mathrm{RI}\left(j_{s}\right)=\left|J_{i}\right|\left(j_{s}\in J_{i},1⩽i⩽k\right)$. In general, back-spliced circRNAs with higher RI are more difficult to reconstruct and quantify.

### Simulation-based validation

In the simulation dataset, 2923 circular transcripts were simulated on the basis of a back-spliced circRNA list from an Hs68 dataset deposited in the NCBI Sequence Read Archive (SRA: SRR444974). Exons between the back splice sites were randomly selected as skipped exons to generate multiple circRNA isoforms, and each isoform was assigned a random expression level based on the actual biological gene expression range. Simulated paired-end reads in the full-length circular transcript sequence were generated at random. Several datasets with different sequencing depths and different read lengths were simulated; 0.09 million 100-bp paired-end reads were first generated as one simulated dataset, and then the sequencing depth was increased 2-, 4-, 8-, 16-, 32-, and 64-fold for the other six datasets. Another simulated dataset including 2.96 million 50-bp paired-end reads was also generated, and the read lengths were changed to 75 bp, 100 bp, 125 bp, and 150 bp with the same sequencing depth for the other four datasets. We also generated datasets for 10 circular isoforms from 4 gene loci (*NOLC1*, *PAFAH1B2*, *DHPS*, and *EXOSC10*) expressed under 8 conditions in humans. Read mapping was performed with Tophat2. Then the circRNA junction site list and the SAM alignment file were used by CircAST with default parameters.

To evaluate the performance of CircAST, the sensitivity and precision were used as in other published studies [31]. Additionally, a single metric $F_{1}$ score was also used, which is the harmonic mean of the sensitivity and precision, according to the formula *F*_1_ = 2 × sensitivity × precision/(sensitivity + precision) and equally favors an increase in sensitivity and precision. Furthermore, Pearson correlation coefficient (PCC) and Spearman correlation coefficient (SCC) between the estimated and simulated abundance of each circular transcript were calculated to evaluate the quantification accuracy.

### Mouse testis circRNA library preparation and sequencing

Total RNA was isolated from the testis tissues of 1-week-old, 3-week-old, or adult mice with an RNeasy Plus Mini Kit (catalog No. 74134, Qiagen, Hilden, Germany) according to the manufacturer’s procedure. The RNA integrity was assessed on an Agilent 2100 Bioanalyzer Lab-on-Chip system (Agilent Technologies, Palo Alto, CA). Approximately 1 µg of total RNA was used to deplete rRNA according to the manufacturer’s instructions for the NEBNext rRNA Depletion Kit (Human/Mouse/Rat) (catalog No. E6310S/E6310L/E6310X; New England Biolabs, Beverly, MA). The rRNA-depleted RNA was further incubated at 37 °C for 10 min with one unit of 20 U/μl RNase R (catalog No. RNR07250; Epicentre, Madison, WI) to digest linear RNA. The remaining RNA was used as templates for the construction of cDNA libraries in accordance with the protocol for the NEBNext® Ultra™ Directional RNA Library Prep Kit for Illumina® (catalog No. E7760S and E7760L; New England Biolabs). The clustering of samples was performed on a cBot Cluster Generation System with a TruSeq PE Cluster Kit v3-cBot-HS (catalog No. PE-401-3001; Illumina, San Diego, CA) according to the manufacturer’s instructions. Paired-end sequencing was performed on the Illumina Hiseq1500 platform. To filter rRNA and mitochondrial RNA (mtRNA), the sequences were aligned against those of the mouse rRNA and mtRNA by using SortmeRNA (version 2.1b), after which the remaining unmapped reads were used for downstream analysis [32].

### Validation of assembled circRNA isoforms by RT-PCR and Sanger sequencing

To validate circRNAs by PCR, RNA without RNase R (Epicentre) treatment and RNA treated with 20 U/μl RNase R were reversely transcribed to cDNA with PrimeScript™ RT Master Mix (Perfect Real Time) (Takara Bio, Otsu, Japan). cDNAs from RNA with or without RNase R treatment, genomic DNA, and negative control (water) were analyzed by PCR amplification for 30 rounds with primers specifically designed for each isoform using a PCR kit (Premix Taq, Takara). Nested PCR was then performed to amplify the weakly abundant isoforms when the specific primers yielded either a negative or a weak result. All primers used in PCR analysis are listed in Table S1. PCR products were detected on a 3% agarose gel stained with ethidium bromide under UV illumination.

Real-time PCR analyses were performed with AceQ qPCR SYBR Green Master Mix (High ROX Premixed) (Vazyme, Nanjing, China) according to the manufacturer’s instructions with a QuantStudio 5 Real-Time PCR System (Thermo Fisher Scientific, Foster City, CA) with the following program: initial denaturation at 95 °C for 5 min, followed by 40 cycles of amplification (denaturation at 95 °C for 10 s and a combined annealing/extension step at 60 °C for 1 min), and a final extension at 72 °C for 5 min. Fold differences were determined by using the comparative threshold cycle method. Circular transcripts of *circCcar* and *circGcl* from *Ccar* and *Gcl* genes were used for normalization. All primers used in the real-time PCR analysis are listed in Table S2.

PCR products were separated by gel electrophoresis, excised, and purified for verification by sequencing. Sequencing was usually performed on both DNA strands, by using both forward and reverse primers. Sequencing reactions were performed in an ABI 2720 Thermal cycler (Applied Biosystems, Foster City, CA). The sequencing products were analyzed with an ABI Hitachi 3730XL DNA Analyzer (Applied Biosystems).

## Results

### Analysis of full-length circRNAs in mouse testis by CircAST

Previous studies have suggested that RNase R can remove linear RNAs and enrich both circular RNAs and intron lariats [33]. Most intronic circular RNAs are derived from intron lariats after the digestion of lariat linear tails with RNase R [25]. Therefore, in this work, we focused on back-spliced exonic circRNAs only. We first performed RNA-seq on adult mouse testis samples after RNase R treatment and RNA library preparation. After mapping using Tophat2, CircAST used the mapped spliced reads to construct multiple splice graphs representing all possible circRNA isoforms for each gene locus, and then used a mathematical model, called extended minimum path cover (EMPC), to search for the minimal set of paths representing circRNA transcripts that can explain all the observed splicing events in a gene locus (details in Methods). After obtaining the circular transcript candidates, CircAST estimated their abundance by using a maximum likelihood estimation algorithm (Figure 1). Finally, we assembled 3464 circRNA full-length transcripts from 2883 back-spliced circRNAs (≥10 supporting back-spliced reads) and estimated their expression abundance in adult mouse testis RNA-seq data (Figure 2A). Of 2803 successfully reconstructed back-spliced circRNAs, approximately 82% produced only one isoform, 14% had two isoforms, and the remaining 4% had three or more isoforms (Figure 2B). We were unable to assemble any transcripts for 80 circRNAs, owing to insufficient forward-spliced reads, possibly because of their low abundance or the false positive BSJs detected by upstream tools. We also found that 17% (593 out of 3464) of these circular transcripts produced distinct AS events, yielding sequences different from those of their linear cognate mRNAs (Figure 2C). We found 380 novel AS events in these 593 circular transcripts (Table S3), thus indicating that circRNAs may use a different splicing mechanism from that of their linear cognate mRNAs.

**Figure 2:**
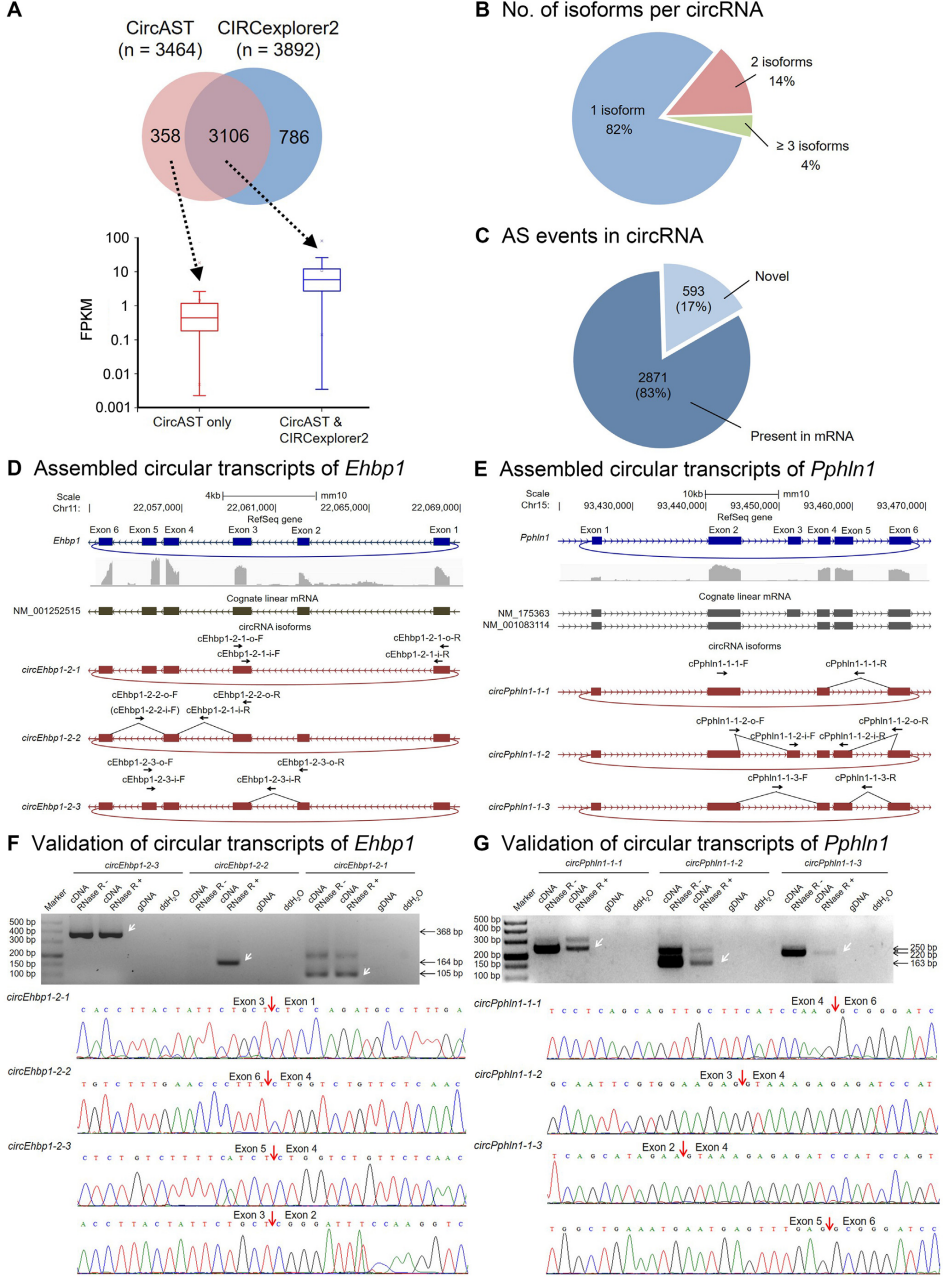
**Circular transcripts assembled by CircAST on adult mouse testis data** **A.** Venn diagram showing a comparison between the circular isoforms reconstructed by CircAST and CIRCexplorer2 from 2883 mouse testis back-spliced circRNAs (No. of supporting reads ≥10) (Top). Box plots of expression of circRNA isoforms from the Venn diagram, quantified by CircAST in FPKM (Bottom). **B.** Distribution of isoform number in each back splicing event. **C.** Comparison of the AS events of circular transcripts with cognate linear transcripts from the same gene locus. **D.** Visualization of assembled circular transcripts in the gene loci *Ehbp1* (Chr11:22,053,432–22,068,506). **E.** Visualization of assembled circular transcripts in the gene loci *Pphln1* (Chr15:93,424,014–93,465,245). For panels D and E, PCR primers are annotated together with the parental gene structure and cognate linear mRNA model. **F.** RT-PCR and Sanger sequencing for all the circular transcripts in the gene loci *Ehbp1* (Chr11:22,053,432–22,068,506). **G.** RT-PCR and Sanger sequencing for all the circular transcripts in the gene loci *Pphln1* (Chr15:93,424,014–93,465,245). For panels F and G, white arrows point to the isoform in the RNase R-treated sample with the correct product size, which was later subjected to Sanger sequencing. There are three isoforms for both circRNAs, in which *circEhbp1-2*-*2* and *circPphln1-1*-*1* were predicted by only CircAST but missed by CIRCexplorer2. The primer sequences are provided in Table S1. AS, alternative splicing.

In addition, we evaluated the percentage of circRNAs assembled on the mouse testis datasets with different circRNA sizes, sequencing depths, read lengths, and library sizes (Figure S2). We found that CircAST performed more efficiently for circRNAs longer than 200 bp. We also found that, although the number of detected back-spliced circRNAs decreased with decreasing read length or sequencing depth, CircAST was still able to assemble a high percentage of circRNAs. The number of detected circRNAs or the percentage of assembled circRNAs in the mouse testis data were comparable with library sizes varied from 250 bp to 300 bp (Figure S2). CircAST captured more circRNA isoforms with library sizes smaller than 400 bp in the brain tissue data (SRA: SRR7350933) (Figure S3). Therefore, the optimal library size for circRNA sequencing is 300 bp on average for CircAST.

We also applied CIRCexplorer2 to the same mouse testis dataset. CIRCexplorer2 assembled 3892 exonic full-length circular transcripts from the same 2883 back-spliced circRNAs described above. Among these, an overlap of 3106 circular transcripts was observed between these two tools. CircAST predicted 358 circular transcripts missed by CIRCexplorer2, most of which had relatively low expression levels (Figure 2A). The relative expression of circRNAs specifically assembled by CIRCexplorer2 could not be calculated because CIRCexplorer2 was not able to quantify circRNA isoforms.

To further validate the predicted circular transcripts, RT-PCR and Sanger sequencing analyses were performed. We randomly selected seven back-spliced circRNAs, each with two or three isoforms identified by CircAST. Of the 16 isoforms of these circRNAs, 13 were successfully validated by both RT-PCR and Sanger sequencing (including two novel isoforms assembled only by CircAST), but three were not detected (Table S4). In addition, we selected three back-spliced circRNAs that have multiple isoforms, each with one isoform identified only by CircAST but missed by CIRCexplorer2. These three isoforms were further subjected to validation, and all were validated by RT-PCR and Sanger sequencing. In total, 19 circular isoforms were selected for validation, and 16 were successfully validated, including five novel isoforms missed by CIRCexplorer2 (Figure 2D–G, Figures S4, and S5). The relative expression levels of different circular isoforms analyzed by RT-PCR were consistent with the computational results (Figure S4B, C).

Because long circRNAs might be difficult to assemble, we chose nine additional long circRNAs (>1200 bp) with two isoforms for experimental validation. All 16 isoforms were successfully validated by both RT-PCR and Sanger sequencing, but validation for two isoforms failed because of usage of unspecific primers (Figure S6).

Since CIRCexplorer2 predicted 786 circRNAs missed by CircAST, we chose eight circRNA isoforms with possible false intron-retention or incorrect use of cassette exons for validation by nested PCR and Sanger sequencing (Figure S7, Table S5). RT-PCR analysis showed that either no amplification product was obtained or size of the product is different from the expected size. Further Sanger sequencing of the incorrectly-sized products further confirmed that the recovered sequences did not contain the expected splicing isoforms. Therefore, all these eight circRNA isoforms were found to be false positive predictions by CIRCexplorer2 and could be treated as true negatives in CircAST calculation.

To evaluate the accuracy of circRNA isoform quantification by CircAST, we performed quantitative real-time PCR (qPCR) analysis of circRNA isoforms in testis samples from 1-week-old and 3-week-old mice. We used *circCcar* and *circGcl* transcripts as internal controls for normalization, due to their similar expression levels across different samples in RNA-seq data. qPCR analysis of eight different genes showed that the quantification of circRNA expression levels by qPCR was consistent with that in RNA-Seq data by CircAST (Figure S8), thus confirming the accuracy of CircAST in circRNA isoform quantification.

### Simulation studies

Current circRNA transcript annotations for many species are not comprehensive, and the existing circRNA databases are far from complete [34], [35]. Therefore, analysis of real data alone is not sufficient to evaluate the performance of computational tools. Nonetheless, simulation can aid in evaluating how well our assembler captures both the full-length structure and quantifies the level of each circular isoform (details in Methods).

On the basis of a BSJ list of 2610 circRNAs from the Hs68 data (Methods), we generated seven simulated paired-end sequencing datasets with fixed read length (100 bp) and variable sequencing depth ranging from 0.09 to 5.92 million reads. As the sequencing depth increased in the simulated data, the sensitivity of CircAST improved steadily, whereas the precision slightly decreased (Figure 3A, top). The $F_{1}$ score reached its best performance at the sequencing depth of 1.48 million. Of note, even at a low sequencing depth of 0.37 million, approximately 86% of circular transcripts were correctly assembled by CircAST. Conversely, even at the highest sequencing depth of 5.92 million, the precision of CircAST was still more than 81%.

**Figure 3:**
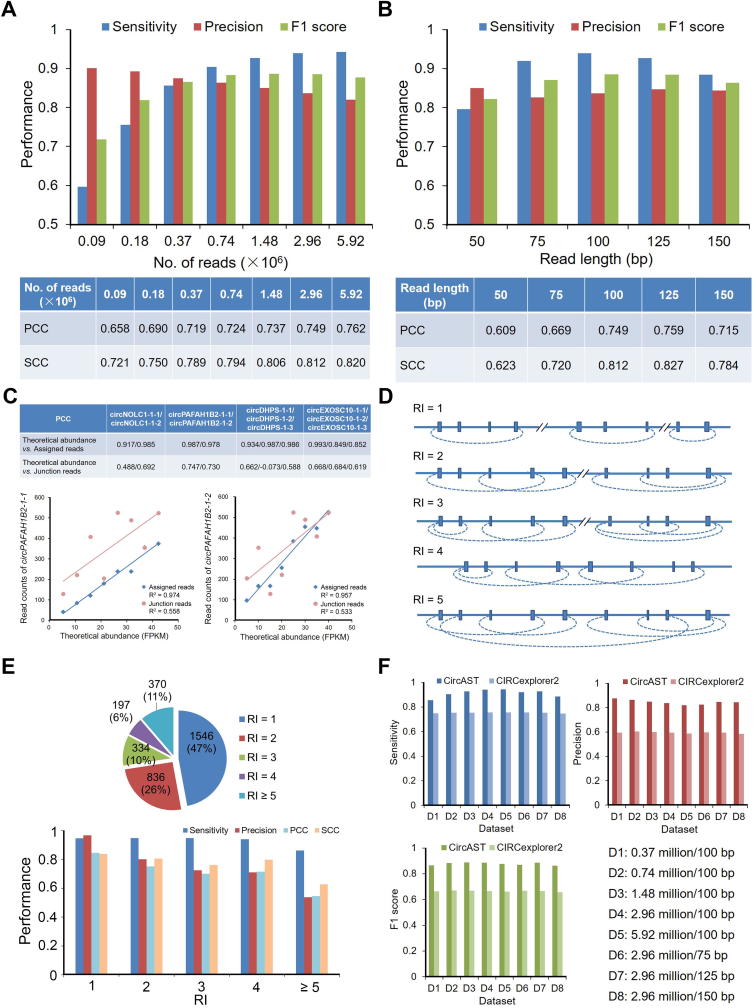
**Performance of CircAST on simulated datasets** **A.** The sensitivity, precision, and $F_{1}$ score of full-length assembly by CircAST (upper), and PCC and SCC (lower) between theoretical and estimated absolute expression levels across different circular transcripts assembled by CircAST in simulated datasets with different sequencing depths. **B.** The sensitivity, precision, and $F_{1}$ score of full-length assembly by CircAST (upper), and Pearson and Spearman correlation coefficients (lower) between theoretical and estimated absolute expression levels across different circular transcripts assembled by CircAST in simulated datasets with different read lengths. **C.** PCC between the theoretical and estimated relative expression levels by CircAST for four back-spliced loci with two or more isoforms due to AS in simulated datasets. Correlation plots between the theoretical abundance and calculated read counts from junction reads or assigned reads by CircAST are shown for *circPAFAH1B2-1*-*1* and *circPAFAH1B2-1*-*2*. **D.** Schematics of RI of equivalence classes to indicate the complexity of circRNAs due to the presence of overlapping regions. **E.** RI distribution of equivalence classes and effects of RI on the accuracy of circular transcript assembly and abundance estimation by CircAST. **F.** Comparison of the sensitivity, precision, and $F_{1}$ score between CircAST and CIRCexplorer2 on simulated datasets with different read lengths and sequencing depths. PCC, Pearson correlation coefficient; SCC, Spearman correlation coefficient; RI, relation index.

To evaluate the accuracy of quantification of circRNA transcript abundance, we computed the PCC and SCC between the actual and estimated expression values for all of the simulated circular transcripts in each set (Figure 3A, bottom). The results showed that both PCC and SCC were above 0.7 in most simulated data and had a tendency to increase when sequencing depth increased, thus suggesting that CircAST performed well in the estimation of absolute abundance.

We next tested the performance of CircAST on variable read lengths with 2.96 million reads. CircAST performed best on read lengths ranging from 75 bp to 125 bp, and it was also able to handle shorter or longer read lengths, such as 50 bp or 150 bp (Figure 3B, top). PCC and SCC showed that absolute quantification performance of CircAST was best on read lengths ranging from 100 to 150 bp (Figure 3B, bottom).

Biologists are usually primarily concerned about the relative quantification levels of certain circRNAs across conditions or samples. To evaluate the relative quantification performance of CircAST for certain circRNAs among different conditions, we simulated dynamic changes of four circRNA loci with two or more splicing isoforms. We found that the correlation coefficients were 0.849–0.993 between the theoretical values and the abundance values estimated by CircAST (Figure 3C). The accuracies obtained by CircAST were far better than those generated using junction reads as a quantification index, which was widely used in the literature [14], [15], [16]. Since junction reads cannot differentiate the expression levels of circRNA isoforms derived from the same back splice site due to AS, it is important and necessary to quantify these isoforms by assigning reads to different circular isoforms according to circular models.

The reconstruction and abundance estimation increases in complexity when circRNAs from multiple BSJ sites have overlapping genomic regions. To evaluate the complexity, we defined a circRNA RI as the cardinality of the equivalence class to which it belongs, that is, $\mathrm{RI}\left(j_{s}\right)=\left|J_{i}\right|\left(j_{s}\in J_{i},1⩽i⩽k\right)$ as shown in Figure 3D (details in Methods). As shown in Figure 3E, when the RI value increased, the precision of circular transcripts reconstruction decreased, and the accuracy of their quantification declined. However, the sensitivity remained relatively stable, which is above 93%, with RI ranging from 1 to 4, and declined to 86%, with RI > 4. These results suggest that the overlapping degree of genomic regions among circRNAs affects the performance of CircAST.

### Comparison with previous methods

We also compared CircAST with CIRCexplorer2 on simulated datasets. On eight simulated datasets with different sequencing depths and read lengths, CircAST exhibited higher sensitivity ranging from 85.63% to 94.32%, and better precision ranging from 81.96% to 87.55% on all the eight simulation datasets under optimal conditions without remnant linear mRNA, and outperformed CIRCexplorer2 (sensitivity ranging from 74.75% to 75.61% and precision ranging from 58.42% to 60.42%) (Figure 3F).

Sailfish-cir can estimate circRNA expression in the same back splice site but cannot quantify each circular isoform. Therefore, we selected circRNAs with only one isoform in the simulated datasets to compare CircAST with Sailfish-cir in circRNA quantification. We calculated PCCs between theoretical and estimated expression levels estimated by the two tools (Table S6). The results showed that CircAST performed better than Sailfish-cir in circRNA quantification.

CIRI-AS is the first efficient tool to detect circRNA internal structure and AS events near the back splice site by using long read sequencing data [26]. We performed CIRI-AS analysis on the simulated dataset with sequencing depth of 2.96 million and read length of 150 bp to compare the results with those of CircAST. In the simulation dataset of 2080 circular transcripts without exon skipping, CIRI-AS predicted the whole cirexons of 1697 isoforms (81.59%), whereas CircAST predicted the whole cirexons of 1881 isoforms (90.43%). For the remaining circular transcripts with 835 AS exons, CIRI-AS detected 348 (41.68%), whereas CircAST detected 762 (91.26%). These results indicate that CircAST can identify more *bona fide* AS events in circRNAs. Therefore, CircAST has advantages in assembling full length circRNAs.

CIRI-full is another highly efficient tool to reconstruct circular transcripts using reverse overlap and BSJ features in long read sequencing data [27]. To compare the performance of CircAST with that of CIRI-full, we tested CircAST using different lengths of sequencing reads (PE100, PE150, PE200, and PE250) trimmed from the PE250 data (SRA: SRR7350933) used by CIRI-full and compared the results with those of CIRI-full. CircAST performs best in PE100 sub-dataset, and was able to reconstruct 61.42% of the circular transcripts in the CIRI-full results by using PE100 data (Figure S9). These data indicate that CircAST has its advantages in assembling long circRNAs without requiring long read sequencing data.

Both CIRI-AS and CIRI-full are annotation-free methods that can capture novel circRNA transcripts including intronic or intergenic circRNAs. CircAST is an annotation-based method, and it can detect only exon-annotated circRNA isoforms. Thus, most of the circular isoforms missed by CircAST were intronic or intergenic. Nonetheless, most of the circular isoforms missed by CIRI-full were longer exonic circular isoforms with length ≥600 bp. These results indicate that CIRI-full can capture circular isoforms with length ≤600 bp using longer read sequencing data, and CircAST is best at identifying circular isoforms of variable length by using short read sequencing data only. Thus these two tools have different advantages in identifying different types of circular isoforms. They can complement each other in reconstructing circular isoforms.

Considering the annotated CIRI-full results as the true circRNA transcripts, we computed a CircAST sensitivity of 80.46% for the trimmed PE100 RNA-seq data and a sensitivity of 76.05% for the trimmed PE150 RNA-seq data. These results indicate that CircAST has better performance in capturing the AS circular isoforms by using short length sequencing reads.

### The diversity of circRNA transcripts in human, mouse, and chicken data

Six datasets were obtained from the NCBI Sequence Read Archive, including five datasets for three human cell lines, HeLa (SRA: SRR1636985, SRR1636986, SRR3476956) [15], HEK293 (SRA: SRR3479244) [26], and Hs68 (SRA: SRR444974) [4], as well as one chicken muscle dataset (SRA: SRR4734704) [36]. We then performed CircAST analysis on these datasets for back-spliced circRNAs with at least five independent supporting reads. In the datasets for three human cell lines, 5253, 4533, and 11,412 exonic circRNA transcripts within 4549, 4201, and 9982 back-spliced circRNAs were built, respectively. There was a high diversity in these circular transcripts among these three cell lines, suggesting that circRNA expression is regulated differentially in different cell types (Figure 4A).

**Figure 4:**
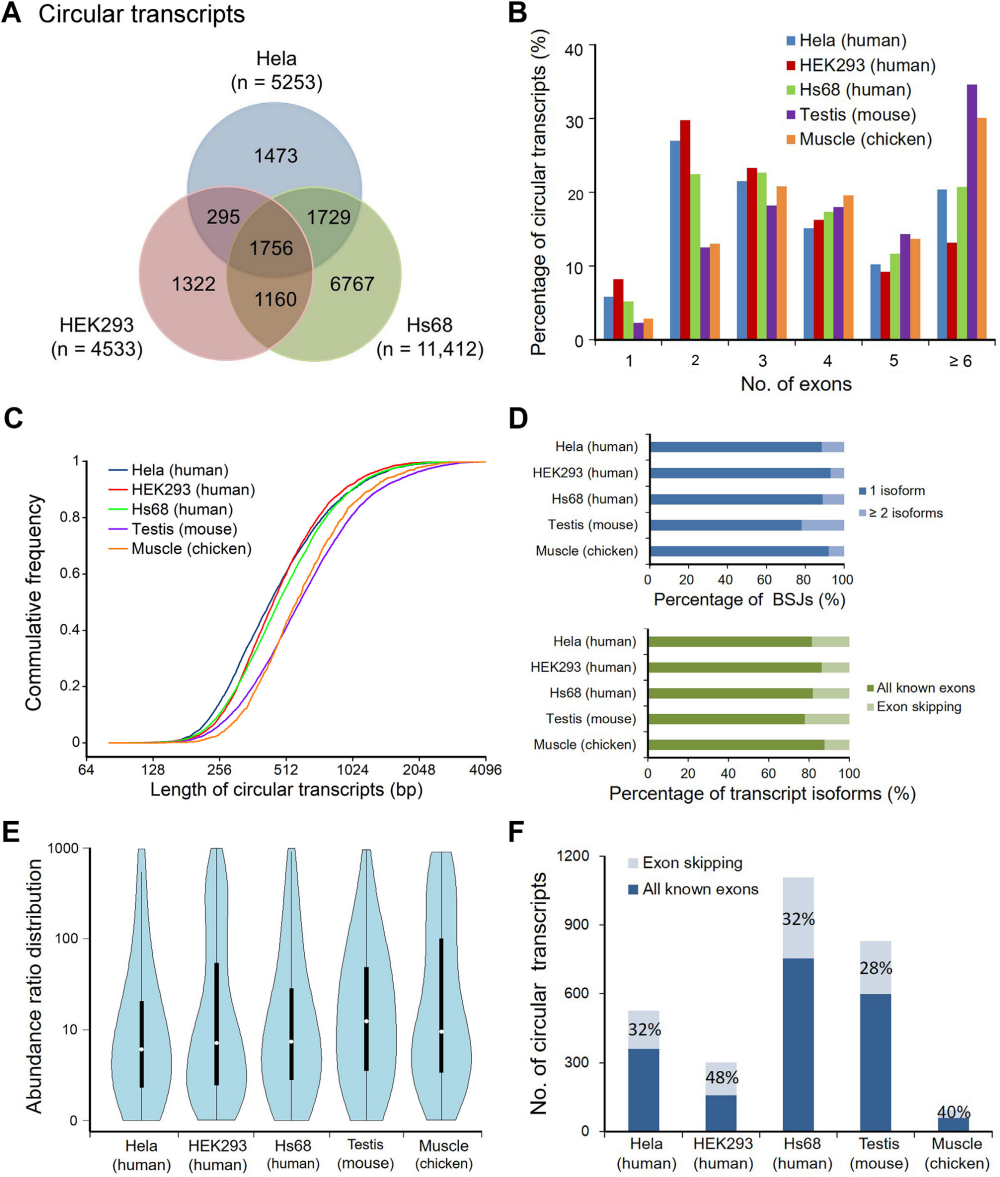
**Analysis of circular transcripts assembled by CircAST in three human cell lines, mouse testis, and chicken muscle** **A.** Venn diagram depicting the diversity of circular transcripts assembled by CircAST among the three human cell lines HeLa, HEK293, and Hs68. **B.** The distribution of exon number in circular transcripts in different cell lines and tissues. **C.** Length distribution of circular transcripts in different cell lines and tissues. **D.** Distribution of circular isoform number in each back splicing event, and the percentage of transcript isoforms containing all known exons between back splice sites in different cell lines and tissues. **E.** The abundance ratio distribution of the top two most abundant circular isoforms from circRNAs with multiple isoforms in each back splice site. **F.** For the most abundant isoform in each circRNA with multiple isoforms, there is a large proportion (28%–48%) of exon skipping events.

In addition, 6452 and 1304 exonic circRNA transcripts within 5362 and 1203 back-spliced circRNAs were also reconstructed from mouse testis (GSA: CRA002302) and chicken muscle data, respectively. We analyzed the genomic and transcriptomic features of the circular transcripts expressed in these datasets and found that in the three human cell lines, most of the assembled exonic circRNA transcripts contained two to four exons, accounting for approximately 60% of circRNA transcripts, whereas 13%–20% of circRNA transcripts contained six or more exons. Interestingly, we found that in mouse testis and chicken muscle samples, circRNA transcripts contained more exons, with ≥6 exons found in >30% transcripts (Figure 4B). Of all the circRNA transcripts, the full length of 50% transcripts is in the range of 100–600 bp, and 10% of them were longer than 1200 bp. On average, the circRNA transcripts in mouse testis and chicken muscle were slightly longer than those found in human cell lines (Figure 4C). This interesting observation was able to be revealed only after the circular isoforms were assembled correctly.

We investigated the AS events in the circRNAs from all datasets. Approximately 7%–22% of back splice sites produced multiple circular isoforms through AS, and approximately 14%–22% of the circular transcripts underwent exon skipping, as shown in Figure 4D. These observations suggest that AS events within circRNAs are widely present in different species, and it is crucial to identify their precise full-length sequences. We also found that some AS events were present only in circRNAs but absent in their linear cognates (Tables S7–S10), which is consistent with the observation in mouse testis (Figure 2C, Table S3).

For circRNAs with multiple isoforms generated by AS, we computed the relative abundance ratio of the top two isoforms. As shown in Figure 4E, abundance ratios of 46.2%–62.2% of the top two isoforms were below 10, indicating that there are at least two predominant isoforms for approximately half of these circRNAs, thereby expanding the diversity of circRNAs. Furthermore, we observed that approximately 28%–48% of these most abundant circular transcripts skipped one or more exons within the back splice sites (Figure 4F). These results together suggest that it is neither appropriate to simply concatenate all known exons between back splice sites sequentially along the genome to derive putative full-length circular transcripts, nor can the most abundant isoform be predicted in this way.

## Discussion

Here, we propose CircAST, a novel downstream computational tool, to assemble and quantify circRNA isoforms from RNase R-treated RNA-seq data after identification of circRNA junction sites. We constructed multiple splice graphs at each gene locus on the basis of the BSJ signatures of sequencing data without relying on any prior assumptions and used the EMPC algorithm on these multiple splice graphs to assemble the multiple circular transcripts. We then used a maximum likelihood estimation algorithm to calculate the expression levels. Extensive simulation studies showed that CircAST has excellent performance in both the assembly and quantification of circular transcripts with regard to sensitivity, precision, and correlation coefficients. The experimental validation results indicate that CircAST can correctly assemble the full-length sequences of circRNAs and quantify their expression, even for weakly abundant circular isoforms, and can reveal many novel AS events in mouse testis tissue.

With CircAST, thousands of exonic circular transcripts were successfully reconstructed. Interestingly they were found to be highly diverse among different cell lines or tissues, thus suggesting that the expression of circRNAs is highly regulated. We found that some AS events were present only for circRNAs but not their cognate linear mRNAs, thus indicating that it is inappropriate to obtain full-length circRNAs by the simple use of linear mRNA counterparts, as performed in previous reports [26], [35]. Our algorithm enables full-length assembly and quantification of circular transcripts simultaneously, and it is directly compatible with commonly used upstream BSJ detection tools (CIRCexplorer2, CIRI2, and UROBORUS). Other circRNA junction detection tools can also be used with CircAST if their output file format is converted to CircAST input file format. We expect that this tool will have important roles in future functional studies of circRNAs.

Although CircAST has many advantages, it has some limitations as compared with previous methods (CIRI-full and CIRI-AS). CircAST can be applied only to RNase R-treated samples, in which linear mRNA may be left over. The remnant linear mRNA in the samples can affect the performance of circular transcript assembly and generate false transcripts to some extent. Thus, careful sample preparation is crucial before using CircAST.

The current version of the CircAST pipeline can assemble full-length sequences and quantify exon-annotated circular transcripts, but may miss intronic or intergenic circular transcripts. Although these intronic or intergenic transcripts account for only a small fraction of the total circular transcripts, we plan to upgrade the CircAST pipeline to reconstruct and quantify intronic circular transcripts or previously uncharacterized exons in the future.

## Availability

Mouse testis rRNA^−^/RNase R-treated RNA-seq data have been deposited in the Genome Sequence Archive (GSA: CRA002302). rRNA−/RNase R-treated RNA-seq data of human cell lines, including Hela, HEK293, and Hs68, chicken muscle tissue, and human brain tissue were downloaded from the NCBI Sequence Reads Archive (SRA: SRR1636985, SRR1636986, SRR3476956; SRR444974; SRR3479244; SRR4734704; SRR7350933).

CircAST can be accessed freely at https://github.com/xiaofengsong/CircAST.

## Authors’ contributions

XS conceived the study and supervised the work. JW designed the method and programed the tool. ZH and XG conceived the experimental validation. YL, Cheng Wang, and YC performed the experimental validation. JW and TX performed the simulation study. JW prepared tables, figures, and legends. XS, XG, and ZH wrote and edited the manuscript. Chang Wang, XW, JS, BJ, and KW assisted with analysis and revised the manuscript. All authors read and approved the final manuscript.

## Competing interests

The authors have declared no competing interests.

## Figures and Tables

**Supplementary Figure S1:**
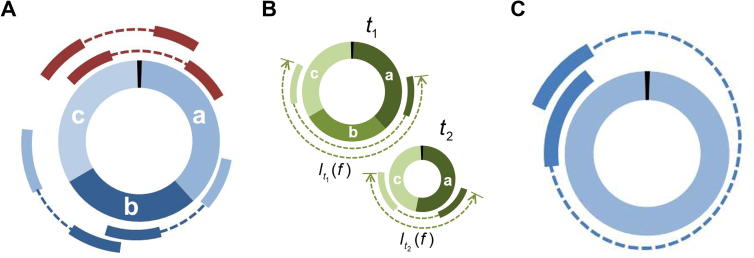
**Schematics of read mapping and fragment length in circRNAs A.** CircAST uses the fragments located not only between the junction sites (blue) but also across the junction (red) in circular transcript assembly and quantification. **B.** Alignments of reads to the genome may be consistent with multiple circular transcripts. The implied length of the fragment *f*, denoted as *l_t_*(*f*), may be different because of the prevalence of AS in circular transcripts. The circular transcripts *t*_1_ and *t*_1_ differ by an internal exon b, with the implied length *l*_t1_(f) > *l*_t2_(*f*). **C.** If the length of a circRNA is shorter than the fragment length, the two reads from the fragment termini could possibly be aligned to the same region in circRNA. a, b, c represent 3 different exons and the site colored in black represents a BSJ.

**Supplementary Figure S2:**
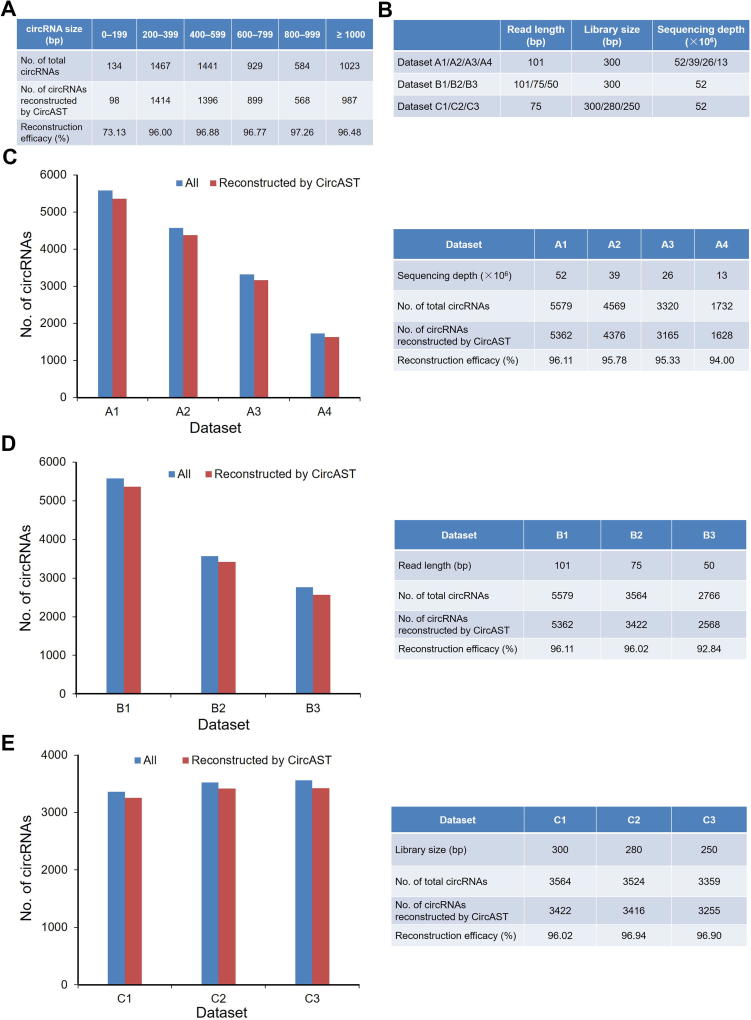
**Performance of CircAST on real mouse testis data with different circRNA sizes, sequencing depths, read lengths, and library sizes A.** Reconstruction efficiency of CircAST with different circRNA sizes. **B.** Datasets on adult mouse testis with different sequencing depths, read lengths, and library sizes. Reconstruction efficiency of CircAST with different sequencing depths **(C)**, read lengths **(D)**, and library sizes **(E)**.

**Supplementary Figure S3:**
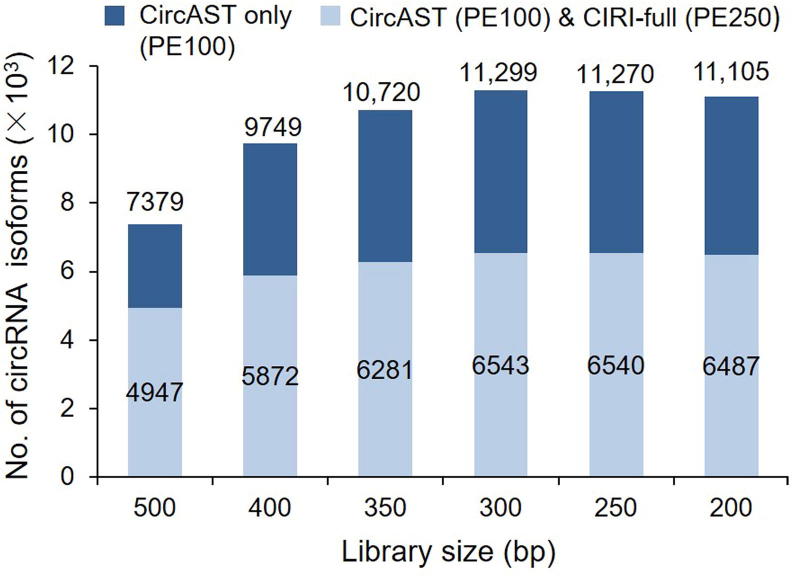
**Performance of CircAST on different library sizes ranging from 500 bp to 200 bp**

**Supplementary Figure S4:**
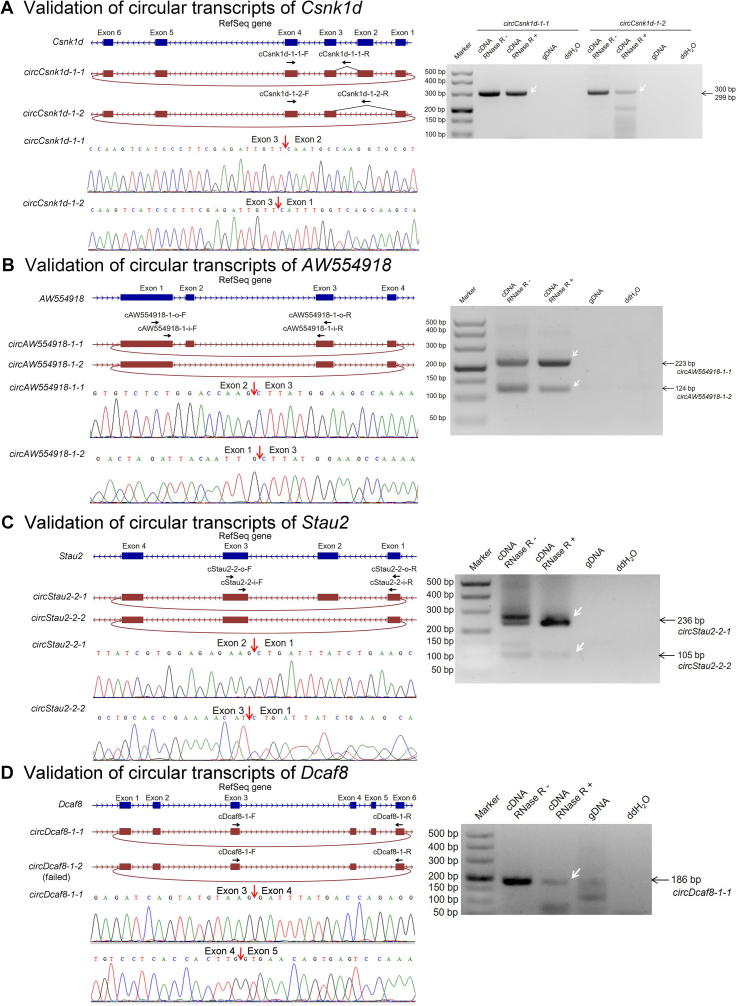
**Validation of all circRNA isoforms at different gene loci detected by CircAST** Schematic models of all circRNA isoforms of *Csnk1d* (Chr11:120,967,995–120,973,969) **(A)**, *AW554918* (Chr18:25,339,714–25,420,075) **(B)**, Stau2 (Chr1:16,440,323–16,509,408) **(C)**, and *Dcaf8* (Chr1:172,173,943–172,187,460) **(D)** are provided separately (top rows on the left). The RT-PCR results (on the right) for each circRNA isoform and the corresponding Sanger sequencing data showing correct specific splicing sites (bottom rows on the left) are provided for validation. All circRNA isoforms in the four gene loci were validated successfully except *circDcaf8-1-2*.

**Supplementary Figure S5:**
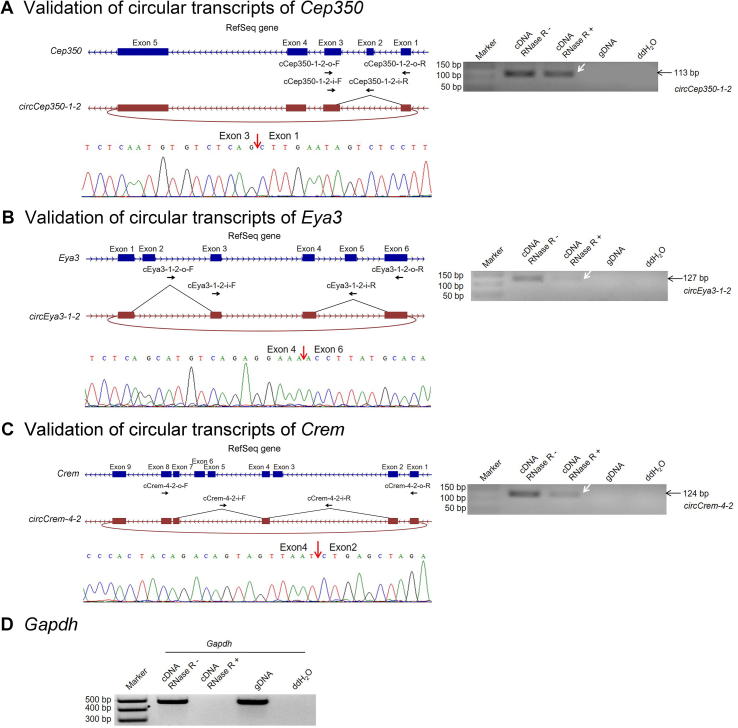
**Validation of the circRNA isoforms at different gene loci detected by CircAST but missed by CIRCexplorer2** Schematic models of circRNA isoforms of *Cep350* (Chr1:155,953,154–155,962,560) **(A)**, Eya3 (Chr4:132,656,693–132,673,032) **(B)**, and *Crem* (Chr18:3,287,904–3,327,591) **(C)** are provided separately (top rows on the left). The RT-PCR results (on the right) for each circRNA isoform and the corresponding Sanger sequencing data showing correct specific splicing sites (bottom rows on the left) are provided for validation. All three isoforms were validated successfully. **D.***Gapdh* was used as a negative control.

**Supplementary Figure S6-1:**
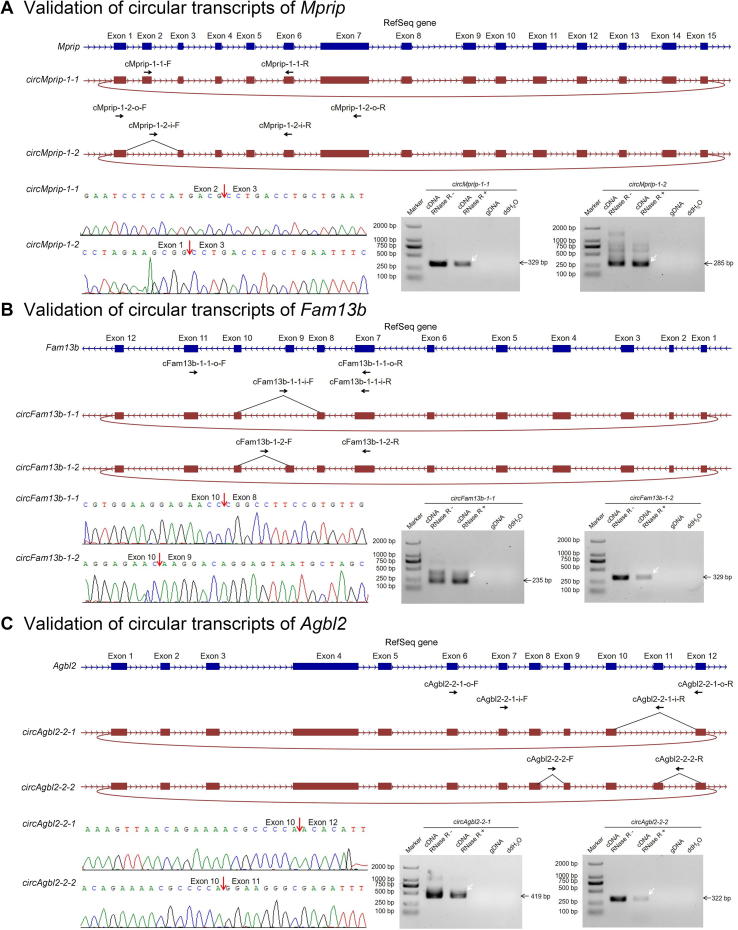
**Validation of the long circRNA isoforms in different gene loci predicted by CircAST** The schematic models of circRNA isoforms of *Mprip* (Chr11: 59,741,120–59,771,702) **(A)**, *Fam13b* (Chr18: 34,443,901–34,465,220) **(B)**, *Agbl2* (Chr2: 90,791,460–90,813,352) **(C)**, *Sbno1* (Chr5: 124,384,467–124,414,527) **(D)**, *Bptf* (Chr11: 107,043,633–107,077,798) **(E)**, *Helz* (Chr11: 107,592,706–107,649,340) **(F)**, *March6* (Chr15: 31,478,295–31,509,822) **(G)**, *Zfp638* (Chr6: 83,934,949–83,972,283) **(H)**, and *Ascc3* (Chr10: 50,690,115–50,767,507) **(I)** are provided separately (top rows). The RT-PCR results for each circRNA isoform and the corresponding Sanger sequencing data showing correct specific splicing sites (bottom rows) are provided for validation. **J.***Gapdh* was used as a negative control.

**Supplementary Figure S6-2:**
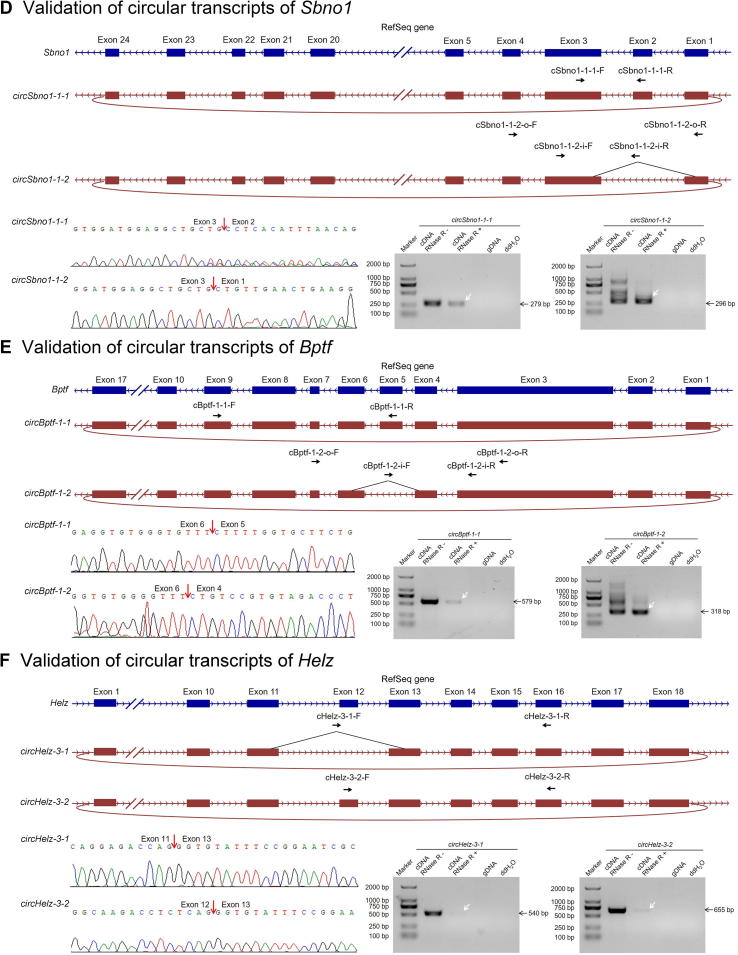


**Supplementary Figure S6-3:**
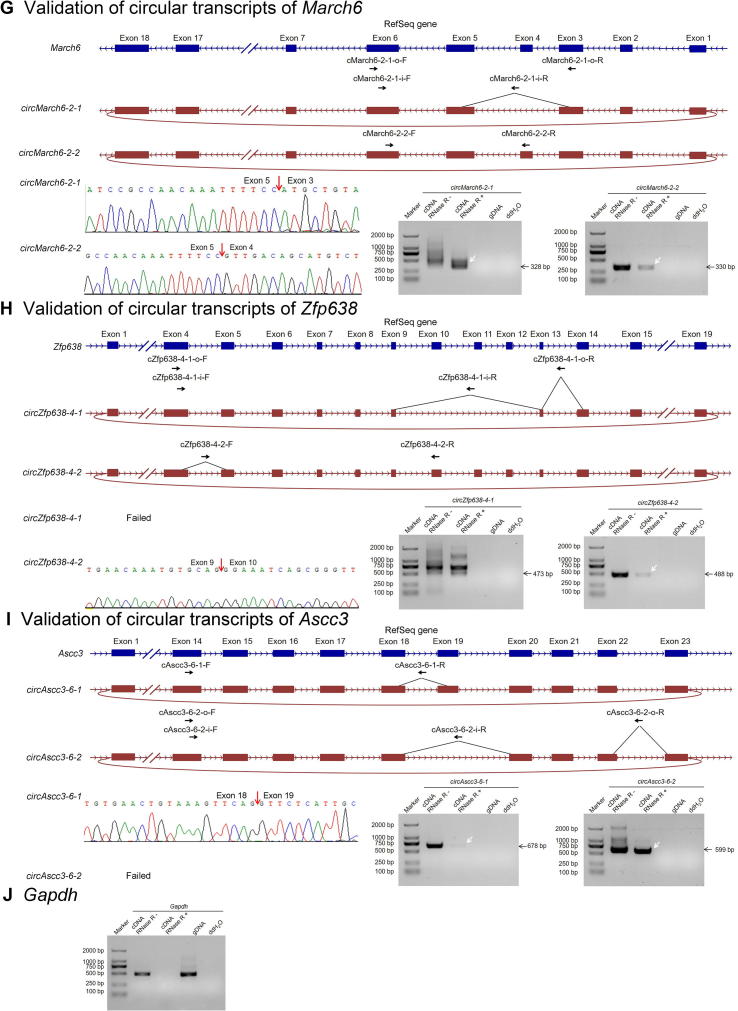


**Supplementary Figure S7:**
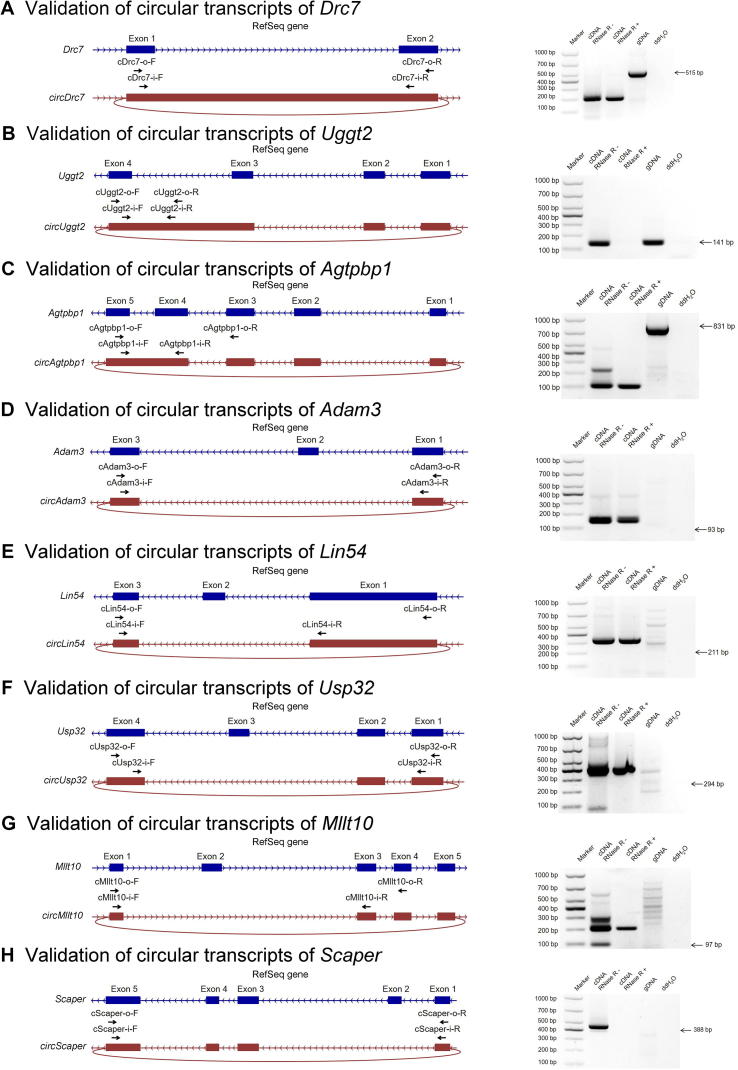
**Validation of the circRNA isoforms in different gene loci predicted by CIRCexplorer2 but missed by CircAST** The schematic models of circRNA isoforms of Drc7 (Chr8:95,061,713–95,062,395) **(A)**, *Uggt2* (Chr14:118,994,946–119,002,986) **(B)**, *Agtpbp1* (Chr13:59,473,688–59,482,604) **(C)**, Adam3 (Chr8:24,719,415–24,725,361) **(D)**, *Lin54* (Chr5:100,475,689–100,485,855) **(E)**, Usp32 (Chr11:85,017,630–85,022,905) **(F)**, *Mllt10* (Chr2:18,101,458–18,126,229) **(G)**, and *Scaper* (Chr9:55,912,040–55,921,338) **(H)** are provided separately (on the left). The RT-PCR results for each circRNA isoform showing incorrect product size are provided separately (on the right). These eight circRNA isoforms failed validation.

**Supplementary Figure S8:**
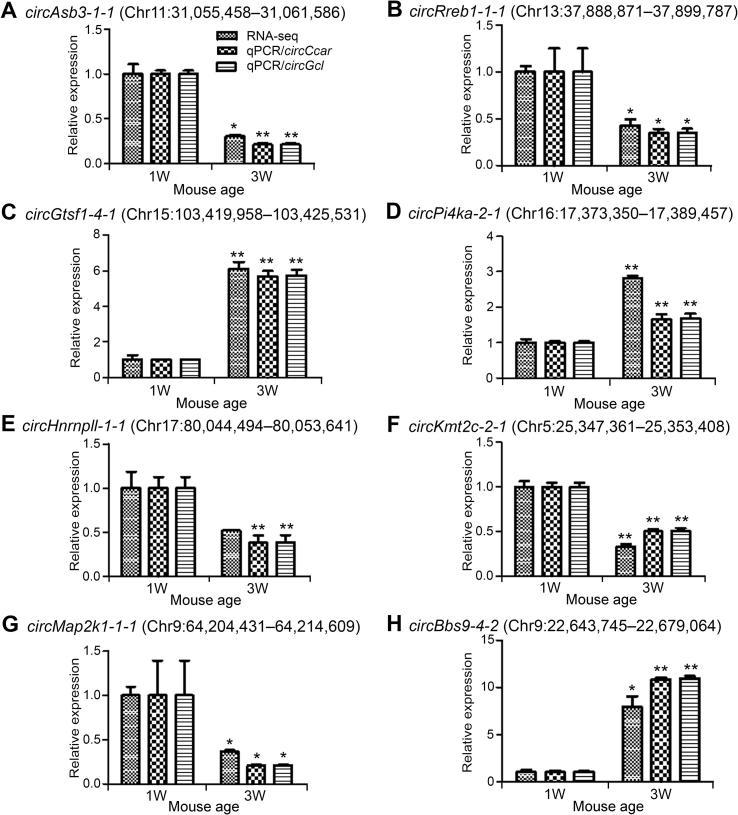
**Quantitative validation of expression of circRNA transcripts calculated by CircAST** Real-time PCR analysis of the expression of circRNAs in testes from 1-week-old and 3-week-old mice was performed with *circCcar* or *circGcl* as internal controls for normalization. FPKM values of the corresponding circRNA transcripts determined by CircAST in RNA-seq data are also given. Expression levels of circAsb3-1-1 **(A)**, *circRreb1-1-1***(B)**, *circGtsf1-4-1***(C)**, *circPi4ka-2-1***(D)**, *circHnrnpll-1-1***(E)**, *circKmt2c-2-1***(F)**, *circMap2k1-1-1***(G)**, and *circBbs9-4-2***(H)** were normalized to those from testes samples of 1-week-old mice. Comparison of circRNA expression between testes samples of 1W and 3W mice was perform by two-tailed independent Student's t-test. *, *P* < 0.05; **, *P* < 0.01.

**Supplementary Figure S9:**
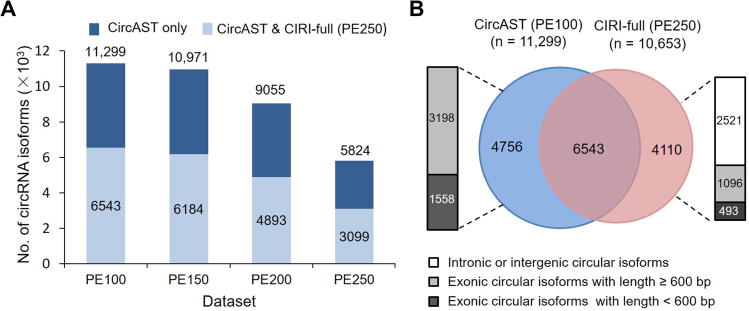
**Performance of CircAST on different lengths of sequencing reads trimmed from PE250 data (SRR7350933) used in CIRI-full A.** CircAST with PE100 reconstructed 61.42% circRNAs of CIRI-full results, and its performance got lower as the sequencing reads became longer; **B.** CircAST missed mostly intronic or intergenic circular isoforms, and CIRI-full missed mostly longer exonic circular isoforms with length ≥ 600 bp. These two methods have different advantages in identifying various types of circular isoforms.
