# Supplementary material for: CircAST: Full-length Assembly and Quantification of Alternatively Spliced Isoforms in Circular RNAs
Source: Genomics Proteomics Bioinformatics. 2020 Jan 31;17(5):522–34. doi: 10.1016/j.gpb.2019.03.004 (PMC7056934; doi:10.1016/j.gpb.2019.03.004)
Supplement: Supplementary Table S1 [file mmc1.docx]

**Table S1 Primer sets used in PCR and nested PCR analyses of mouse testis samples**

| **circRNA isoform name** | **Primer name** | **Primer type** | **Primer sequence (5′-3′)** | **Expected product size (bp)** |
| --- | --- | --- | --- | --- |
| *circEhbp1-2-1* | cEhbp1-2-1-o-F | outer-F | TTTCTCCTTGCTCGGAGGC | (238) |
|  | cEhbp1-2-1-o-R | outer-R | GAGAGGGCTCGTCAGCTAAT |  |
|  | cEhbp1-2-1-i-F | inner-F | TCCACCACCTTACTATTCTG | 105 |
|  | cEhbp1-2-1-i-R | inner-R | ATGCGGAGTGAAGATGTC |  |
| *circEhbp1-2-2* | cEhbp1-2-2-o-F | outer-F | TGTCTTTGAACCCTTTCTGGTCT | (222) |
|  | cEhbp1-2-2-o-R | outer-R | GGTGGCAAACTTACTCCCCA |  |
|  | cEhbp1-2-2-i-F | inner-F | TGTCTTTGAACCCTTTCTGGTCT | 164 |
|  | cEhbp1-2-2-i-R | inner-R | GGAGAAAAGAGTGGCGTGGA |  |
| *circEhbp1-2-3* | cEhbp1-2-3-o-F | outer-F | TCTTCTGATGGCTTCCTCTGA | (422) |
|  | cEhbp1-2-3-o-R | outer-R | GAAGGCTTTGTTGTAGGAGGTG |  |
|  | cEhbp1-2-3-i-F | inner-F | TGGCTTCCTCTGAGTTTCAGTT | 368 |
|  | cEhbp1-2-3-i-R | inner-R | GTGACCTTGATAATCCCGAGC |  |
| *circPphln1-1-1* | cPphln1-1-1-F | F | CGAGAGATCGGTCTCCCCAT | 250 |
|  | cPphln1-1-1-R | R | GATCCCGCCTTGGATGAAGC |  |
| *circPphln1-1-2* | cPphln1-1-2-o-F | outer-F | CATAGAAAGTCCTCGCGTGTC | (270) |
|  | cPphln1-1-2-o-R | outer-R | TCCCGCCTCAAACTCATTCA |  |
|  | cPphln1-1-2-i-F | inner-F | ACGACAGAATGAAGCAATTCGTG | 163 |
|  | cPphln1-1-2-i-R | inner-R | GCTTTCAGCCTCAGCAAGTTC |  |
| *circPphln1-1-3* | cPphln1-1-3-F | F | TCAGCATAGAAGTAAAGAGAGATCC | 220 |
|  | cPphln1-1-3-R | R | GGATCCCGCCTCAAACTCAT |  |
| *circCsnk1d-1-1* | cCsnk1d-1-1-F | F | GAAGCCTTGGCGATGGAACA | 299 |
|  | cCsnk1d-1-1-R | R | ACACGCACCTTGGCATTGAA |  |
| *circCsnk1d-1-2* | cCsnk1d-1-2-F | F | GAGAAGCCTTGGCGATGGAA | 300 |
|  | cCsnk1d-1-2-R | R | TGCTTGCTGACCAAATGAACAAT |  |
| *circAW554918-1-1/ circAW554918-1-2* | cAW554918-1-o-F | outer-F | AAAAGGAACGCCTTCAGCATC | (294, 195) |
|  | cAW554918-1-o-R | outer-R | TCTTCTCCCTCTGGTTTTGCC |  |
|  | cAW554918-1-i-F | inner-F | CTCCTGGCCCAACAAGAGAC | 223, 124 |
|  | cAW554918-1-i-R | inner-R | AGGATGTTGAGGTTCCACGC |  |
| *circStau2-2-1/ circStau2-2-2* | cStau2-2-o-F | outer-F | CAACAGCTTGTTGGGCCTTC | (276, 145) |
|  | cStau2-2-o-R | outer-R | GTGGAGCTGTGAGGGATACG |  |
|  | cStau2-2-i-F | inner-F | TCGGATTCCCATGTCTGCTC | 236, 105 |
|  | cStau2-2-i-R | inner-R | GCTGTGAGGGATACGGAAGTT |  |
| *circDcaf8/ circDcaf8-1-2* | cDcaf8-1-F | F | TTTGCAGTGGGTGGAAGAGAT | 186, 128 (failed) |
|  | cDcaf8-1-R | R | ATCATTGTAACTGGCCAGGAG |  |
| *circTtc3-1-1/ circTtc3-1-2* | cTtc3-1-F | F | TAGCGATGGAAAGAGGGCCA | 272 (failed), 109 (failed) |
|  | cTtc3-1-R | R | TTCTAGGTATGTAAGCCCTGCT |  |
| *circCep350-1-2* | cCep350-1-2-o-F | outer-F | AGGTTCACGGAACTCCACAC | (323) |
|  | cCep350-1-2-o-R | outer-R | CAGCAAGTCAAAAGAGGTGCC |  |
|  | cCep350-1-2-i-F | inner-F | CGGGTAGCACTTGCAGACTT | 113 |
|  | cCep350-1-2-i-R | inner-R | GCAAGGAGACTATTCAAGCTGAG |  |
| *circEya3-1-2* | cEya3-1-2-o-F | outer-F | ACCAGAGCAACCGGTGAAAA | (254) |
|  | cEya3-1-2-o-R | outer-R | GTTTGGGTTGCCTGAGGGTA |  |
|  | cEya3-1-2-i-F | inner-F | GCCAAGATGCAGGAACCAAG | 127 |
|  | cEya3-1-2-i-R | inner-R | TGTGCATAAGGTTTTCCTCTGAC |  |
| *circCrem-4-2* | cCrem-4-2-o-F | outer-F | TGTATTGCCCCGTGCTAGTC | (510) |
|  | cCrem-4-2-o-R | outer-R | ATGAGCAAATGTGGCAGGAA |  |
|  | cCrem-4-2-i-F | inner-F | GCTACCTTTTTATCCAGATCCCCT | 124 |
|  | cCrem-4-2-i-R | inner-R | CTAGCTCAGATTAACTACTGTCTGT |  |
| *GAPDH-Con* | GAPDH-Con-F | F | AGTGGCAAAGTGGAGATTGTT | 488 |
|  | GAPDH-Con-R | R | GTCTTCTGGGTGGCAGTGAT |  |
| *circDrc7* | cDrc7-o-F | outer-F | GGGACCTGACCAGCAAGTTT | 571 |
|  | cDrc7-o-R | outer-R | GCTCTCAATGCCCAGGAAGT |  |
|  | cDrc7-i-F | inner-F | GCAAGTTTGAGCAGGAGCAA | 515 |
|  | cDrc7-i-R | inner-R | GTGCAGTCAGAGGGTCGATG |  |
| *circUggt2* | cUggt2-o-F | outer-F | ATGTGACCCATTCTGGGACG | 172 |
|  | cUggt2-o-R | outer-R | GTGACATACGACGTTCCCCA |  |
|  | cUggt2-i-F | inner-F | TTGCAGCAGCTTCTAGTTTGG | 141 |
|  | cUggt2-i-R | inner-R | ACGACGTTCCCCAAGAGTTTC |  |
| *circAgtpbp1* | cAgtpbp1-o-F | outer-F | CTCCACTTAAGGAGCAGCGG | 964 |
|  | cAgtpbp1-o-R | outer-R | ACAGCAATGCCAGAGTCCAA |  |
|  | cAgtpbp1-i-F | inner-F | CACTTAAGGAGCAGCGGTGA | 831 |
|  | cAgtpbp1-i-R | inner-R | ACCTCATGAGCAATAGCCCG |  |
| *circAdam3* | cAdam3-o-F | outer-F | AATGTTCCCAACTTGTCACGC | 129 |
|  | cAdam3-o-R | outer-R | ACACCACTTCTGCAGATCACA |  |
|  | cAdam3-i-F | inner-F | TCCCAACTTGTCACGCAAAT | 93 |
|  | cAdam3-i-R | inner-R | AAATCGACACCAACATCCAGG |  |
| *circLin54* | cLin54-o-F | outer-F | TTGGCGACTTCAAAGGTGAG | 810 |
|  | cLin54-o-R | outer-R | CCGGCCGAGGTGAATAGTTT |  |
|  | cLin54-i-F | inner-F | TGGCGACTTCAAAGGTGAGAT | 211 |
|  | cLin54-i-R | inner-R | CCCCAGGAAGCCAACTGATT |  |
| *circUsp32* | cUsp32-o-F | outer-F | AGAGGCGGCATATGACCATT | 416 |
|  | cUsp32-o-R | outer-R | CAAATGCAAGACATGTGGGC |  |
|  | cUsp32-i-F | inner-F | GTCGAGGCAGATCCCCATTG | 294 |
|  | cUsp32-i-R | inner-R | AGTGTCCGATTCGACCCTTTT |  |
| *circMllt10* | cMllt10-o-F | outer-F | GATGTGAGCTGTGTCCCCAT | 204 |
|  | cMllt10-o-R | outer-R | TGCGCCATTCCCTTCTTCTT |  |
|  | cMllt10-i-F | inner-F | TGTGAGCTGTGTCCCCATAA | 97 |
|  | cMllt10-i-R | inner-R | GGCTGCTTTGCTTTCTCGTC |  |
| *circScaper* | cScaper-o-F | outer-F | TTTCATCTACTGCCCGGCG | 401 |
|  | cScaper-o-R | outer-R | ATACCACCGTGGATTTTGGGA |  |
|  | cScaper-i-F | inner-F | CCCGGCGAAGATTGTCAAAA | 388 |
|  | cScaper-i-R | inner-R | TACCACCGTGGATTTTGGGAG |  |
| *circMprip-1-1* | cMprip-1-1-F | F | TCCACACCGAAGAGCCAAGTCA | 329 |
|  | cMprip-1-1-R | R | TCCAGTTCCGTCGTATGCCAGAT |  |
| *circMprip-1-2* | cMprip-1-2-o-F | outer-F | TGCTGATTCTGACCACTCCAT | (849) |
|  | cMprip-1-2-o-R | outer-R | GATGCCAACGCTGCTCAATT |  |
|  | cMprip-1-2-i-F | inner-F | ATCCCTAGAAAGCGGCCTGA | 285 |
|  | cMprip-1-2-i-R | inner-R | GTTCCGTCGTATGCCAGATGT |  |
| *circFam13b-1-1* | cFam13b-1-1-o-F | outer-F | CAGAGGAGAGGCTGACACCATCTT | (339) |
|  | cFam13b-1-1-o-R | outer-R | ATGAGGACGGCGAGAGTGAAGG |  |
|  | cFam13b-1-1-i-F | inner-F | TTCGTGGAAGGAGAACCC | 235 |
|  | cFam13b-1-1-i-R | inner-R | AGGACGGCGAGAGTGAA |  |
| *circFam13b-1-2* | cFam13b-1-2-F | F | GGAAGGAGAACCAAGGACAGGAGT | 329 |
|  | cFam13b-1-2-R | R | ATGAGGACGGCGAGAGTGAAGG |  |
| *circAgbl2-2-1* | cAgbl2-2-1-o-F | outer-F | GAAGAGTGGTGATGTGGCGGATG | (560) |
|  | cAgbl2-2-1-o-R | outer-R | CTTAGGGCTGGTCAGTGGTGGAT |  |
|  | cAgbl2-2-1-i-F | inner-F | TTACCATTGAGGACCTGAAG | 419 |
|  | cAgbl2-2-1-i-R | inner-R | TCCACTTAACTGTGTTGGG |  |
| *circAgbl2-2-2* | cAgbl2-2-2-F | F | ACATTGAATCCAGCACGAGTG | 322 |
|  | cAgbl2-2-2-R | R | AACTGTGTCTCATTGAGCCTTG |  |
| *circSbno1-1-1* | cSbno1-1-1-F | F | GAGCAGCGGGCATGGCATTT | 279 |
|  | cSbno1-1-1-R | R | GGTTGGAGACAGAAGCAGCAGTTC |  |
| *circSbno1-1-2* | cSbno1-1-2-o-F | outer-F | TTATCCATATCCTCGCCACTTCTT | (487) |
|  | cSbno1-1-2-o-R | outer-R | TTACTGCTTGCTGCCTTGAGT |  |
|  | cSbno1-1-2-i-F | inner-F | GCGGGTGGCTTCAGTTTCAT | 296 |
|  | cSbno1-1-2-i-R | inner-R | CCTTCAGTTCAACAGCAGCAG |  |
| *circBptf-1-1* | cBptf-1-1-F | F | TTGCTGGCTTGGACCTGTAG | 579 |
|  | cBptf-1-1-R | R | CCTTATGGCATTCGTTCTGAGTAT |  |
| *circBptf-1-2* | cBptf-1-2-o-F | outer-F | TCTGCTGCTCTGCTGCTTGA | (934) |
|  | cBptf-1-2-o-R | outer-R | ATGGATGACAATGGACTGCCTTC |  |
|  | cBptf-1-2-i-F | inner-F | GAGGTGTGGGTGTTTCTGTCCGTG | 318 |
|  | cBptf-1-2-i-R | inner-R | GGAAGTTCGTTACCAAGAGCAGCA |  |
| *circHelz-3-1* | cHelz-3-1-F | F | CAGCAGGAGACCAGGGTGTAT | 540 |
|  | cHelz-3-1-R | R | CGTGAGTGGATAGAAGTCCTTGTG |  |
| *circHelz-3-2* | cHelz-3-2-F | F | TCTAATAGTGCTGCTGACCTCTAC | 655 |
|  | cHelz-3-2-R | R | CCGTGCTGTTCTTCTCTTGGA |  |
| *circMarch6-2-1* | cMarch6-2-1-o-F | outer-F | GCATCAGCAGCATCTTCTACAC | (469) |
|  | cMarch6-2-1-o-R | outer-R | CCTTCACGGCTACCAATCCAA |  |
|  | cMarch6-2-1-i-F | inner-F | TCCTCCTCATTGTCCTCCTCTT | 328 |
|  | cMarch6-2-1-i-R | inner-R | GTTGTTCCTCTTACAGCATGGAAA |  |
| *circMarch6-2-2* | cMarch6-2-2-F | F | TCCTCTTCCTCTTCTGCTTGAC | 330 |
|  | cMarch6-2-2-R | R | CTCACTACTGACACTGCCACTA |  |
| *circZfp638-4-1* | cZfp638-4-1-o-F | outer-F | GGCATTAGAAGATGGAGGACAACG | (743) |
|  | cZfp638-4-1-o-R | outer-R | CCGATTCCTTGTTCACTGGTTCC |  |
|  | cZfp638-4-1-i-F | inner-F | AAGTGCTTTGGCCCAGCGGAAG | 473 (failed) |
|  | cZfp638-4-1-i-R | inner-R | CTGTGGCCTCCAAAGTACCTGCA |  |
| *circZfp638-4-2* | cZfp638-4-2-F | F | CCAGCGGAAGCCACAGAAGGAT | 488 |
|  | cZfp638-4-2-R | R | TCGCAGCTACCGTAACCACAGATT |  |
| *circAscc3-6-1* | cAscc3-6-1-F | F | GACCTTATTGTCACCACACCAGAA | 678 |
|  | cAscc3-6-1-R | R | GCTTGTAGCAATGAGAACCTGAAC |  |
| *circAscc3-6-2* | cAscc3-6-2-o-F | outer-F | GACCTTATTGTCACCACACCAGAA | 1042 |
|  | cAscc3-6-2-o-R | outer-R | GTGCGATTATCCTCTCCAACTTCA |  |
|  | cAscc3-6-2-i-F | inner-F | CAGCAGGTCAACATTCTCATC | 599 (failed) |
|  | cAscc3-6-2-i-R | inner-R | CAGCACATCTGAACTTTACAGT |  |
