# Supplementary material for: CircAST: Full-length Assembly and Quantification of Alternatively Spliced Isoforms in Circular RNAs
Source: Genomics Proteomics Bioinformatics. 2020 Jan 31;17(5):522–34. doi: 10.1016/j.gpb.2019.03.004 (PMC7056934; doi:10.1016/j.gpb.2019.03.004)
Supplement: Supplementary Table S2 [file mmc2.docx]

**Table S2 Primer sets used in qPCR**

| **circRNA isoform name** | **Primer names** | **Primer sequences (5′-3′)** |
| --- | --- | --- |
| *circAsb3-1-1* | cAsb3-1-1-F | GCTGCTGCACAAATGGGCCATACA |
|  | cAsb3-1-1-R | GGCACTCTTCCCGTCCTCCAAACA |
| *circRreb1-1-1* | cRreb1-1-1-F | CGTAGCGAGTGTCACAGAGAA |
|  | cRreb1-1-1-R | TGTGTTGTGCTGACGGATGT |
| *circGtsf1-4-1* | cGtsf1-4-1-F | CCAGAGTCTCTTGTCCAAGGTTCC |
|  | cGtsf1-4-1-R | TTGGCTACTTGTCCCTTCAATGCT |
| *circPi4ka-2-1* | cPi4ka-2-1-F | TGGGCTAACCTGAGAGATGCTGGA |
|  | cPi4ka-2-1-R | CTAGAAGGTGTCCGAAGGCGTTCC |
| *circHnrnpll-1-1* | cHnrnpll-1-1-F | GCTTGTCTCTGGCGACCCTTTCCT |
|  | cHnrnpll-1-1-R | GCCCAGAAGGCTAAAGCAGCACTC |
| *circKmt2c-2-1* | cKmt2c-2-1-F | GCTGTGACTGTGAGGCTCTGTAG |
|  | cKmt2c-2-1-R | GACTGCTGCGACTCTTCTCTTGT |
| *circMap2k1-1-1* | cMap2k1-1-1-F | ATCTCGCCGTCGCTGTAGAA |
|  | cMap2k1-1-1-R | GGTGGAGTGGTCTTCAAGGTCT |
| *circBbs9-4-2* | cBbs9-4-2-F | CCTGTCAGTCTGCTCGGTCTCTTC |
|  | cBbs9-4-2-R | AGGCGGAGGATGAGTTCATTGGTT |
| *circCcar* | cCcar-F | ACTGAAGACTCCGACTGCTGTTAT |
|  | cCcar-R | CTGTGGCTGCGTCTGCAATAG |
| *circGcl* | cGcl-F | GGCGATGTTCTTGAGACTCTG |
|  | cGcl-R | CTCCACAGTGTTGAACTCAGAC |
