# Supplementary material for: CircAST: Full-length Assembly and Quantification of Alternatively Spliced Isoforms in Circular RNAs
Source: Genomics Proteomics Bioinformatics. 2020 Jan 31;17(5):522–34. doi: 10.1016/j.gpb.2019.03.004 (PMC7056934; doi:10.1016/j.gpb.2019.03.004)
Supplement: Supplementary Table S3 [file mmc3.docx]

**Table S3 Novel AS events in circular transcripts from adult mouse testis samples supported by ≥ 2 solid junction reads**

| **Chr** | **Location of**  **5' donor site** | **Location of**  **3' acceptor site** | **No. of forward splice junction reads** |
| --- | --- | --- | --- |
| Chr10 | 125,294,844 | 125,301,957 | 1324 |
| Chr5 | 134,622,086 | 134,625,366 | 410 |
| Chr1 | 10,000,137 | 10,003,235 | 243 |
| Chr15 | 10,587,924 | 10,591,587 | 211 |
| Chr14 | 54,667,161 | 54,679,241 | 161 |
| Chr12 | 84,425,280 | 84,428,343 | 150 |
| Chr3 | 95,785,106 | 95,787,284 | 141 |
| Chr6 | 39,648,486 | 39,651,605 | 133 |
| Chr6 | 39,644,380 | 39,648,450 | 132 |
| Chr11 | 22,059,261 | 22,068,359 | 124 |
| Chr2 | 18,170,298 | 18,186,218 | 124 |
| Chr16 | 14,582,616 | 14,589,188 | 123 |
| Chr16 | 94,385,423 | 94,388,265 | 118 |
| Chr11 | 79,458,906 | 79,468,717 | 116 |
| Chr10 | 123,153,127 | 123,168,227 | 105 |
| Chr13 | 59,528,518 | 59,532,013 | 105 |
| Chr2 | 68,696,528 | 68,711,021 | 98 |
| Chr15 | 36,010,337 | 36,010,742 | 97 |
| Chr3 | 65,725,430 | 65,739,682 | 95 |
| Chr17 | 13,823,458 | 13,828,929 | 91 |
| Chr1 | 5,098,133 | 5,117,390 | 90 |
| Chr12 | 8,699,188 | 8,705,933 | 85 |
| Chr1 | 84,776,412 | 84,782,827 | 80 |
| Chr2 | 140,008,819 | 140,048,242 | 80 |
| Chr7 | 29,204,922 | 29,206,805 | 80 |
| Chr10 | 108,271,769 | 108,274,138 | 79 |
| Chr11 | 54,482,586 | 54,489,269 | 77 |
| Chr14 | 31,044,709 | 31,046,332 | 68 |
| Chr5 | 138,283,152 | 138,290,709 | 63 |
| Chr9 | 44,158,891 | 44,163,339 | 60 |
| Chr12 | 11,274,111 | 11,279,605 | 59 |
| Chr1 | 10,083,558 | 10,088,051 | 57 |
| Chr11 | 97,376,541 | 97,377,936 | 57 |
| Chr11 | 5,620,147 | 5,630,702 | 53 |
| Chr17 | 56,653,756 | 56,655,097 | 52 |
| Chr7 | 102,163,822 | 102,176,313 | 52 |
| Chr14 | 31,054,255 | 31,061,469 | 51 |
| Chr13 | 44,970,178 | 44,986,035 | 50 |
| Chr19 | 45,761,208 | 45,765,450 | 47 |
| Chr8 | 92,346,918 | 92,350,691 | 46 |
| Chr12 | 84,423,503 | 84,428,343 | 45 |
| Chr6 | 83,954,761 | 83,958,184 | 43 |
| Chr18 | 25,340,384 | 25,399,926 | 41 |
| Chr4 | 70,290,029 | 70,298,820 | 39 |
| Chr16 | 94,403,368 | 94,409,684 | 38 |
| Chr7 | 120,278,327 | 120,283,019 | 37 |
| Chr19 | 32,223,590 | 32,289,541 | 36 |
| Chr1 | 46,083,813 | 46,097,998 | 35 |
| Chr1 | 161,845,358 | 161,847,205 | 34 |
| Chr3 | 146,469,803 | 146,476,194 | 33 |
| Chr9 | 102,619,760 | 102,623,556 | 33 |
| Chr17 | 63,941,757 | 63,973,065 | 32 |
| Chr11 | 104,357,596 | 104,378,685 | 31 |
| Chr6 | 85,643,283 | 85,656,395 | 31 |
| Chr2 | 120,783,799 | 120,790,282 | 30 |
| Chr16 | 94,384,066 | 94,384,784 | 29 |
| Chr2 | 121,321,792 | 121,327,645 | 29 |
| Chr2 | 152,302,337 | 152,307,989 | 29 |
| Chr12 | 108,158,348 | 108,162,973 | 28 |
| Chr15 | 10,583,836 | 10,591,587 | 28 |
| Chr10 | 60,007,826 | 60,013,596 | 27 |
| Chr11 | 70,917,782 | 70,920,852 | 27 |
| Chr13 | 92,468,162 | 92,477,828 | 27 |
| Chr14 | 14,859,359 | 14,860,926 | 26 |
| Chr5 | 5,452,067 | 5,457,285 | 26 |
| Chr6 | 92,149,804 | 92,154,335 | 26 |
| Chr6 | 71,894,372 | 71,895,905 | 26 |
| Chr10 | 85,903,012 | 85,905,349 | 25 |
| Chr11 | 82,722,483 | 82,736,314 | 25 |
| Chr17 | 25,167,816 | 25,168,310 | 25 |
| Chr13 | 8,820,545 | 8,836,783 | 23 |
| Chr15 | 39,437,922 | 39,454,360 | 23 |
| Chr17 | 13,849,662 | 13,852,316 | 23 |
| Chr3 | 65,739,867 | 65,781,371 | 23 |
| Chr5 | 28,339,308 | 28,352,472 | 23 |
| Chr5 | 32,878,677 | 32,893,375 | 23 |
| Chr14 | 47,500,870 | 47,504,986 | 22 |
| Chr17 | 63,941,480 | 63,957,125 | 22 |
| Chr5 | 41,687,850 | 41,703,438 | 22 |
| Chr5 | 5,640,227 | 5,646,936 | 22 |
| Chr12 | 101,510,525 | 101,541,408 | 21 |
| Chr2 | 121,321,792 | 121,331,558 | 21 |
| Chr2 | 18,071,204 | 18,109,782 | 21 |
| Chr4 | 109,840,442 | 109,842,288 | 21 |
| Chr11 | 97,373,130 | 97,377,936 | 20 |
| Chr17 | 88,671,619 | 88,694,103 | 20 |
| Chr2 | 68,307,108 | 68,314,364 | 20 |
| Chr10 | 58,507,641 | 58,513,079 | 19 |
| Chr1 | 88,228,408 | 88,230,626 | 18 |
| Chr4 | 32,709,566 | 32,711,941 | 18 |
| Chr4 | 111,780,842 | 111,799,070 | 18 |
| Chr5 | 67,315,845 | 67,326,853 | 18 |
| Chr2 | 166,936,026 | 166,939,635 | 17 |
| Chr2 | 49,530,143 | 49,535,255 | 17 |
| Chr4 | 137,635,008 | 137,637,760 | 17 |
| Chr5 | 92,633,421 | 92,636,191 | 17 |
| Chr11 | 97,241,043 | 97,241,951 | 16 |
| Chr17 | 30,708,526 | 30,712,273 | 16 |
| Chr17 | 26,739,668 | 26,757,805 | 16 |
| Chr18 | 3,309,755 | 3,325,361 | 16 |
| Chr18 | 34,494,776 | 34,499,686 | 16 |
| Chr2 | 5,877,001 | 5,882,581 | 16 |
| Chr7 | 29,204,922 | 29,206,554 | 16 |
| Chr9 | 23,301,022 | 23,373,833 | 16 |
| Chr12 | 101,415,369 | 101,446,026 | 15 |
| Chr17 | 30,706,511 | 30,712,273 | 15 |
| Chr18 | 34,497,546 | 34,499,686 | 15 |
| Chr2 | 5,877,001 | 5,882,818 | 15 |
| Chr9 | 55,807,120 | 55,809,102 | 15 |
| Chr1 | 176,826,284 | 176,831,057 | 14 |
| Chr12 | 101,509,326 | 101,541,408 | 14 |
| Chr12 | 3,632,964 | 3,648,223 | 14 |
| Chr14 | 33,605,084 | 33,612,296 | 14 |
| Chr3 | 102,853,498 | 102,864,857 | 14 |
| Chr3 | 105,917,098 | 105,919,975 | 14 |
| Chr4 | 150,500,051 | 150,534,847 | 14 |
| Chr4 | 103,319,528 | 103,325,019 | 14 |
| Chr5 | 34,716,329 | 34,730,005 | 14 |
| Chr9 | 107,025,381 | 107,055,450 | 14 |
| Chr9 | 55,858,525 | 55,859,748 | 14 |
| Chr10 | 41,501,931 | 41,507,424 | 13 |
| Chr17 | 30,704,851 | 30,708,291 | 13 |
| Chr2 | 49,018,325 | 49,099,717 | 13 |
| Chr2 | 166,930,666 | 166,934,753 | 13 |
| Chr2 | 6,394,555 | 6,408,929 | 13 |
| Chr6 | 90,633,755 | 90,636,857 | 13 |
| Chr7 | 92,614,552 | 92,623,968 | 13 |
| Chr1 | 55,323,721 | 55,346,377 | 12 |
| Chr11 | 74,945,632 | 74,981,852 | 12 |
| Chr12 | 72,906,279 | 72,909,904 | 12 |
| Chr2 | 29,901,247 | 29,903,321 | 12 |
| Chr2 | 29,150,107 | 29,156,988 | 12 |
| Chr4 | 83,546,969 | 83,554,694 | 12 |
| Chr4 | 108,650,346 | 108,660,565 | 12 |
| Chr7 | 109,312,420 | 109,321,461 | 12 |
| Chr7 | 14,598,451 | 14,607,240 | 12 |
| Chr1 | 98,490,650 | 98,497,477 | 11 |
| Chr12 | 86,256,932 | 86,261,616 | 11 |
| Chr13 | 59,473,817 | 59,475,660 | 11 |
| Chr14 | 86,866,642 | 86,910,029 | 11 |
| Chr14 | 57,441,982 | 57,445,565 | 11 |
| Chr14 | 60,239,546 | 60,242,547 | 11 |
| Chr15 | 74,625,557 | 74,626,278 | 11 |
| Chr2 | 30,296,702 | 30,300,800 | 11 |
| Chr3 | 7,526,417 | 7,539,076 | 11 |
| Chr5 | 53,883,694 | 53,889,316 | 11 |
| Chr7 | 29,719,587 | 29,721,267 | 11 |
| Chr11 | 87,140,601 | 87,145,464 | 10 |
| Chr14 | 33,624,989 | 33,632,906 | 10 |
| Chr17 | 50,935,558 | 51,143,359 | 10 |
| Chr3 | 100,644,621 | 100,652,539 | 10 |
| Chr5 | 107,790,508 | 107,797,813 | 10 |
| Chr5 | 32,651,755 | 32,653,157 | 10 |
| Chr7 | 133,868,199 | 133,870,022 | 10 |
| Chr9 | 22,659,145 | 22,678,912 | 10 |
| Chr9 | 59,889,555 | 59,894,761 | 10 |
| Chr9 | 59,907,476 | 59,909,816 | 10 |
| Chr1 | 66,762,252 | 66,773,422 | 9 |
| Chr13 | 59,533,239 | 59,544,387 | 9 |
| Chr14 | 33,612,393 | 33,622,482 | 9 |
| Chr16 | 43,569,781 | 43,577,030 | 9 |
| Chr17 | 64,625,451 | 64,651,196 | 9 |
| Chr17 | 68,457,333 | 68,461,451 | 9 |
| Chr17 | 71,558,679 | 71,569,017 | 9 |
| Chr4 | 127,104,421 | 127,106,576 | 9 |
| Chr5 | 36,523,009 | 36,543,177 | 9 |
| Chr7 | 128,443,946 | 128,444,613 | 9 |
| Chr8 | 77,384,796 | 77,409,548 | 9 |
| Chr1 | 98,446,822 | 98,490,497 | 8 |
| Chr1 | 55,346,452 | 55,355,648 | 8 |
| Chr10 | 83,297,300 | 83,304,304 | 8 |
| Chr11 | 82,739,705 | 82,742,010 | 8 |
| Chr12 | 85,878,478 | 85,889,159 | 8 |
| Chr12 | 11,271,871 | 11,279,605 | 8 |
| Chr12 | 81,895,191 | 81,912,665 | 8 |
| Chr14 | 56,460,124 | 56,465,627 | 8 |
| Chr17 | 30,653,409 | 30,656,886 | 8 |
| Chr3 | 41,682,372 | 41,709,469 | 8 |
| Chr5 | 23,986,814 | 23,991,736 | 8 |
| Chr5 | 92,633,421 | 92,641,011 | 8 |
| Chr6 | 11,987,274 | 11,988,722 | 8 |
| Chr7 | 92,605,380 | 92,612,743 | 8 |
| Chr7 | 56,087,440 | 56,090,915 | 8 |
| Chr8 | 24,872,761 | 24,884,438 | 8 |
| Chr9 | 115,266,193 | 115,276,704 | 8 |
| Chr1 | 46,109,340 | 46,119,260 | 7 |
| Chr1 | 46,081,497 | 46,085,555 | 7 |
| Chr1 | 88,228,408 | 88,233,317 | 7 |
| Chr1 | 84,786,196 | 84,814,823 | 7 |
| Chr1 | 53,627,112 | 53,631,466 | 7 |
| Chr1 | 38,071,497 | 38,079,290 | 7 |
| Chr12 | 77,277,455 | 77,331,896 | 7 |
| Chr12 | 85,879,475 | 85,891,021 | 7 |
| Chr15 | 93,455,530 | 93,465,105 | 7 |
| Chr17 | 30,704,851 | 30,712,273 | 7 |
| Chr17 | 30,636,076 | 30,644,557 | 7 |
| Chr19 | 27,830,792 | 27,849,711 | 7 |
| Chr2 | 37,627,526 | 37,640,851 | 7 |
| Chr3 | 41,667,182 | 41,675,328 | 7 |
| Chr4 | 126,082,985 | 126,084,393 | 7 |
| Chr4 | 141,483,825 | 141,487,566 | 7 |
| Chr5 | 43,188,943 | 43,191,294 | 7 |
| Chr5 | 124,299,079 | 124,306,953 | 7 |
| Chr6 | 120,412,510 | 120,418,559 | 7 |
| Chr7 | 82,268,352 | 82,273,861 | 7 |
| Chr1 | 119,722,036 | 119,741,355 | 6 |
| Chr1 | 87,365,279 | 87,378,972 | 6 |
| Chr10 | 93,846,454 | 93,850,150 | 6 |
| Chr11 | 106,800,143 | 106,804,993 | 6 |
| Chr11 | 120,972,547 | 120,973,821 | 6 |
| Chr12 | 101,481,073 | 101,507,909 | 6 |
| Chr12 | 110,545,901 | 110,552,785 | 6 |
| Chr14 | 56,506,007 | 56,509,279 | 6 |
| Chr14 | 56,431,649 | 56,434,597 | 6 |
| Chr16 | 55,860,379 | 55,890,582 | 6 |
| Chr17 | 30,653,409 | 30,657,969 | 6 |
| Chr2 | 139,996,157 | 140,042,094 | 6 |
| Chr2 | 18,308,949 | 18,316,899 | 6 |
| Chr2 | 166,929,023 | 166,930,582 | 6 |
| Chr2 | 37,624,590 | 37,626,482 | 6 |
| Chr2 | 37,624,590 | 37,627,435 | 6 |
| Chr2 | 147,025,306 | 147,026,515 | 6 |
| Chr3 | 139,243,185 | 139,306,144 | 6 |
| Chr3 | 65,684,106 | 65,781,371 | 6 |
| Chr4 | 152,237,766 | 152,260,846 | 6 |
| Chr4 | 119,339,873 | 119,353,070 | 6 |
| Chr5 | 147,526,680 | 147,527,129 | 6 |
| Chr5 | 34,730,042 | 34,745,163 | 6 |
| Chr6 | 116,284,815 | 116,305,617 | 6 |
| Chr7 | 120,991,910 | 120,998,901 | 6 |
| Chr7 | 82,276,285 | 82,285,021 | 6 |
| Chr7 | 120,283,241 | 120,294,116 | 6 |
| Chr8 | 24,804,544 | 24,810,001 | 6 |
| Chr8 | 77,426,431 | 77,450,693 | 6 |
| Chr9 | 102,603,075 | 102,613,295 | 6 |
| Chr9 | 109,999,840 | 110,027,679 | 6 |
| Chr1 | 177,067,333 | 177,080,201 | 5 |
| Chr1 | 155,959,831 | 155,962,475 | 5 |
| Chr10 | 50,748,943 | 50,750,453 | 5 |
| Chr10 | 123,146,938 | 123,168,227 | 5 |
| Chr10 | 63,016,542 | 63,017,524 | 5 |
| Chr13 | 97,091,952 | 97,094,062 | 5 |
| Chr13 | 94,493,741 | 94,528,165 | 5 |
| Chr13 | 119,386,667 | 119,394,611 | 5 |
| Chr14 | 27,448,260 | 27,449,946 | 5 |
| Chr15 | 36,013,173 | 36,025,808 | 5 |
| Chr15 | 36,025,987 | 36,043,205 | 5 |
| Chr16 | 37,247,948 | 37,261,884 | 5 |
| Chr17 | 86,099,295 | 86,109,227 | 5 |
| Chr17 | 68,559,863 | 68,630,185 | 5 |
| Chr2 | 118,222,460 | 118,236,914 | 5 |
| Chr2 | 126,121,081 | 126,124,580 | 5 |
| Chr2 | 166,932,194 | 166,937,342 | 5 |
| Chr2 | 25,029,656 | 25,039,603 | 5 |
| Chr3 | 75,061,655 | 75,078,388 | 5 |
| Chr3 | 37,083,353 | 37,091,860 | 5 |
| Chr4 | 143,170,188 | 143,180,877 | 5 |
| Chr4 | 21,859,307 | 21,861,987 | 5 |
| Chr5 | 23,985,840 | 23,986,734 | 5 |
| Chr5 | 53,860,206 | 53,874,887 | 5 |
| Chr7 | 109,331,607 | 109,338,320 | 5 |
| Chr8 | 39,067,762 | 39,096,925 | 5 |
| Chr8 | 24,863,545 | 24,870,686 | 5 |
| Chr8 | 111,972,191 | 111,979,299 | 5 |
| Chr8 | 24,815,166 | 24,818,096 | 5 |
| Chr1 | 37,801,905 | 37,806,937 | 4 |
| Chr1 | 160,702,460 | 160,722,133 | 4 |
| Chr1 | 160,682,182 | 160,710,147 | 4 |
| Chr10 | 62,763,523 | 62,765,267 | 4 |
| Chr11 | 79,438,848 | 79,441,891 | 4 |
| Chr11 | 108,506,657 | 108,518,679 | 4 |
| Chr12 | 101,575,978 | 101,594,158 | 4 |
| Chr12 | 101,575,978 | 101,602,675 | 4 |
| Chr12 | 101,509,326 | 101,520,503 | 4 |
| Chr12 | 8,709,509 | 8,713,360 | 4 |
| Chr13 | 63,156,735 | 63,190,479 | 4 |
| Chr16 | 46,448,968 | 46,458,114 | 4 |
| Chr16 | 94,419,478 | 94,422,227 | 4 |
| Chr17 | 26,759,949 | 26,763,326 | 4 |
| Chr17 | 26,736,983 | 26,742,042 | 4 |
| Chr18 | 80,844,602 | 80,858,544 | 4 |
| Chr18 | 35,269,710 | 35,325,284 | 4 |
| Chr19 | 16,710,427 | 16,720,329 | 4 |
| Chr19 | 47,801,979 | 47,813,928 | 4 |
| Chr2 | 121,336,840 | 121,337,449 | 4 |
| Chr2 | 112,347,073 | 112,349,276 | 4 |
| Chr2 | 24,858,250 | 24,863,772 | 4 |
| Chr2 | 28,709,976 | 28,716,417 | 4 |
| Chr2 | 68,694,021 | 68,711,021 | 4 |
| Chr3 | 65,684,106 | 65,739,682 | 4 |
| Chr3 | 56,086,284 | 56,087,397 | 4 |
| Chr5 | 73,510,956 | 73,520,116 | 4 |
| Chr5 | 134,287,043 | 134,293,747 | 4 |
| Chr5 | 92,645,700 | 92,649,936 | 4 |
| Chr6 | 116,273,872 | 116,287,982 | 4 |
| Chr6 | 35,211,017 | 35,214,318 | 4 |
| Chr7 | 127,519,832 | 127,521,989 | 4 |
| Chr8 | 24,637,261 | 24,646,283 | 4 |
| Chr8 | 75,577,994 | 75,629,714 | 4 |
| Chr8 | 33,322,368 | 33,329,102 | 4 |
| Chr8 | 33,334,245 | 33,342,977 | 4 |
| Chr9 | 6,967,289 | 6,992,524 | 4 |
| Chr9 | 59,396,611 | 59,405,725 | 4 |
| Chr9 | 121,044,871 | 121,081,564 | 4 |
| Chr1 | 98,423,349 | 98,432,233 | 3 |
| Chr1 | 37,804,739 | 37,810,972 | 3 |
| Chr1 | 172,187,460 | 172,192,518 | 3 |
| Chr1 | 165,390,533 | 165,422,856 | 3 |
| Chr1 | 9,678,505 | 9,683,097 | 3 |
| Chr1 | 100,383,620 | 100,431,716 | 3 |
| Chr1 | 36,343,384 | 36,349,402 | 3 |
| Chr10 | 88,213,712 | 88,219,250 | 3 |
| Chr11 | 93,921,484 | 93,923,791 | 3 |
| Chr11 | 79,446,922 | 79,448,125 | 3 |
| Chr12 | 31,617,180 | 31,626,726 | 3 |
| Chr12 | 4,344,502 | 4,416,671 | 3 |
| Chr13 | 59,528,518 | 59,533,172 | 3 |
| Chr14 | 56,438,908 | 56,459,966 | 3 |
| Chr14 | 56,460,124 | 56,463,024 | 3 |
| Chr15 | 38,494,366 | 38,497,264 | 3 |
| Chr17 | 84,310,055 | 84,336,793 | 3 |
| Chr17 | 79,861,479 | 79,865,042 | 3 |
| Chr18 | 3,288,092 | 3,299,162 | 3 |
| Chr19 | 36,585,533 | 36,597,077 | 3 |
| Chr2 | 18,123,812 | 18,146,777 | 3 |
| Chr2 | 28,739,664 | 28,759,942 | 3 |
| Chr2 | 154,517,962 | 154,523,904 | 3 |
| Chr3 | 84,864,011 | 84,903,501 | 3 |
| Chr3 | 40,654,253 | 40,672,571 | 3 |
| Chr3 | 7,528,129 | 7,539,076 | 3 |
| Chr4 | 70,298,948 | 70,302,084 | 3 |
| Chr4 | 132,667,234 | 132,672,896 | 3 |
| Chr5 | 36,520,561 | 36,543,177 | 3 |
| Chr5 | 36,525,048 | 36,571,296 | 3 |
| Chr5 | 64,278,044 | 64,285,945 | 3 |
| Chr6 | 38,440,547 | 38,461,475 | 3 |
| Chr6 | 120,374,987 | 120,382,917 | 3 |
| Chr7 | 139,454,095 | 139,511,408 | 3 |
| Chr7 | 122,114,470 | 122,121,054 | 3 |
| Chr7 | 82,268,352 | 82,276,127 | 3 |
| Chr8 | 78,531,158 | 78,662,987 | 3 |
| Chr8 | 111,979,444 | 111,987,122 | 3 |
| Chr8 | 129,136,649 | 129,145,519 | 3 |
| Chr9 | 6,994,146 | 7,001,341 | 3 |
| Chr9 | 102,602,548 | 102,613,295 | 3 |
| Chr9 | 64,311,979 | 64,314,553 | 3 |
| Chr1 | 46,081,497 | 46,097,998 | 2 |
| Chr1 | 172,186,368 | 172,187,353 | 2 |
| Chr1 | 36,346,029 | 36,349,402 | 2 |
| Chr10 | 89,789,381 | 89,791,363 | 2 |
| Chr10 | 41,951,746 | 41,966,701 | 2 |
| Chr10 | 88,111,774 | 88,124,808 | 2 |
| Chr11 | 22,053,555 | 22,056,783 | 2 |
| Chr12 | 51,627,880 | 51,647,994 | 2 |
| Chr12 | 85,876,592 | 85,879,368 | 2 |
| Chr12 | 101,481,073 | 101,541,408 | 2 |
| Chr13 | 44,970,178 | 44,992,019 | 2 |
| Chr13 | 119,389,281 | 119,396,747 | 2 |
| Chr14 | 86,835,101 | 86,905,937 | 2 |
| Chr14 | 20,523,856 | 20,524,357 | 2 |
| Chr16 | 94,384,934 | 94,387,337 | 2 |
| Chr18 | 80,877,373 | 80,909,592 | 2 |
| Chr19 | 3,392,261 | 3,395,920 | 2 |
| Chr2 | 121,348,848 | 121,349,475 | 2 |
| Chr2 | 12,397,566 | 12,403,998 | 2 |
| Chr2 | 139,977,713 | 140,042,094 | 2 |
| Chr2 | 140,008,819 | 140,057,287 | 2 |
| Chr3 | 146,481,860 | 146,487,512 | 2 |
| Chr3 | 86,417,996 | 86,532,085 | 2 |
| Chr3 | 65,725,430 | 65,781,371 | 2 |
| Chr3 | 68,985,483 | 68,990,742 | 2 |
| Chr4 | 138,097,359 | 138,105,314 | 2 |
| Chr6 | 37,903,651 | 37,915,165 | 2 |
| Chr6 | 87,057,764 | 87,060,521 | 2 |
| Chr7 | 109,312,420 | 109,325,681 | 2 |
| Chr7 | 97,636,469 | 97,653,053 | 2 |
| Chr8 | 39,063,308 | 39,071,376 | 2 |
| Chr9 | 6,941,704 | 6,967,080 | 2 |
| Chr9 | 96,566,122 | 96,569,020 | 2 |
| Chr9 | 24,631,279 | 24,645,062 | 2 |
