# Supplementary material for: CircAST: Full-length Assembly and Quantification of Alternatively Spliced Isoforms in Circular RNAs
Source: Genomics Proteomics Bioinformatics. 2020 Jan 31;17(5):522–34. doi: 10.1016/j.gpb.2019.03.004 (PMC7056934; doi:10.1016/j.gpb.2019.03.004)
Supplement: Supplementary Table S4 [file mmc4.docx]

**Table S4 CircAST-assembled circRNA isoforms that were selected for RT-PCR and Sanger sequencing validation**

| **Gene** | **Location of circRNA** | **No. of isoforms predicted by CircAST** | **Isoforms selected for validation** | **Validated by RT-PCR and Sanger sequencing** | **Also predicted by CIRCexplorer2** |
| --- | --- | --- | --- | --- | --- |
| *Ehbp1* | Chr11:22,053,432–22,068,506 | 3 | *circEhbp1-2-1* | Yes | Yes |
|  |  |  | *circEhbp1-2-2* | Yes | No |
|  |  |  | *circEhbp1-2-3* | Yes | Yes |
| *Pphln1* | Chr15:93,424,014–93,465,245 | 3 | *circPphln1-1-1* | Yes | No |
|  |  |  | *circPphln1-1-2* | Yes | Yes |
|  |  |  | *circPphln1-1-3* | Yes | Yes |
| *Csnk1d* | Chr11:120,967,995–120,973,969 | 2 | *circCsnk1d-1-1* | Yes | Yes |
|  |  |  | *circCsnk1d-1-2* | Yes | Yes |
| *AW554918* | Chr18:25,339,714–25,420,075 | 2 | *circAW554918-1-1* | Yes | Yes |
|  |  |  | *circAW554918-1-2* | Yes | Yes |
| *Stau2* | Chr1:16,440,323–16,509,408 | 2 | *circStau2-2-1* | Yes | Yes |
|  |  |  | *circStau2-2-2* | Yes | Yes |
| *Dcaf8* | Chr1:172,173,943–172,187,460 | 2 | *circDcaf8-1-1* | Yes | Yes |
|  |  |  | *circDcaf8-1-2* | No | Yes |
| *Ttc3* | Chr16:94,403,314–94,412,123 | 2 | *circTtc3-1-1* | No | Yes |
|  |  |  | *circTtc3-1-2* | No | Yes |
| *Cep350* | Chr1:155,953,154–155,962,560 | 2 | *circCep350-1-2* | Yes | No |
| *Eya3* | Chr4:132,656,693–132,673,032 | 2 | *circEya3-1-2* | Yes | No |
| *Crem* | Chr18:3,287,904–3,327,591 | 6 | *circCrem-4-2* | Yes | No |
