# Supplementary material for: CircAST: Full-length Assembly and Quantification of Alternatively Spliced Isoforms in Circular RNAs
Source: Genomics Proteomics Bioinformatics. 2020 Jan 31;17(5):522–34. doi: 10.1016/j.gpb.2019.03.004 (PMC7056934; doi:10.1016/j.gpb.2019.03.004)
Supplement: Supplementary Table S5 [file mmc5.docx]

**Table S5 CIRCexplorer2-assembled circRNA isoforms that were selected for RT-PCR and Sanger sequencing validation**

| **Gene** | **Location of circRNA** | **Isoforms selected for validation** | **Predicted by CIRCexplorer2** | **Also predicted by CircAST** | **Validated by RT-PCR and Sanger sequencing** |
| --- | --- | --- | --- | --- | --- |
| *Drc7* | Chr8:95,061,713–95,062,395 | *circDrc7* | Yes | No | No |
| *Uggt2* | Chr14:118,994,946–119,002,986 | *circUggt2* | Yes | No | No |
| *Agtpbp1* | Chr13:59,473,688–59,482,604 | *circAgtpbp1* | Yes | No | No |
| *Adam3* | Chr8:24,719,415–24,725,361 | *circAdam3* | Yes | No | No |
| *Lin54* | Chr5:100,475,689–100,485,855 | *circLin54* | Yes | No | No |
| *Usp32* | Chr11:85,017,630–85,022,905 | *circUsp32* | Yes | No | No |
| *Mllt10* | Chr2:18,101,458–18,126,229 | *circMllt10* | Yes | No | No |
| *Scaper* | Chr9:55,912,040–55,921,338 | *circScaper* | Yes | No | No |
