# Supplementary material for: CircAST: Full-length Assembly and Quantification of Alternatively Spliced Isoforms in Circular RNAs
Source: Genomics Proteomics Bioinformatics. 2020 Jan 31;17(5):522–34. doi: 10.1016/j.gpb.2019.03.004 (PMC7056934; doi:10.1016/j.gpb.2019.03.004)
Supplement: Supplementary Table S6 [file mmc6.docx]

**Table S6 Correlation coefficients between theoretical and estimated expression levels by CircAST and Sailfish-cir**

| **Coefficient** | **Method** | **Sequencing depth (million)** | | | | | | |  | **Read length (bp)** | | | | |
| --- | --- | --- | --- | --- | --- | --- | --- | --- | --- | --- | --- | --- | --- | --- |
|  |  | **0.09** | **0.18** | **0.37** | **0.74** | **1.48** | **2.96** | **5.92** |  | **50** | **75** | **100** | **125** | **150** |
| PCC | CircAST | 0.701 | 0.775 | 0.796 | 0.801 | 0.803 | 0.807 | 0.775 |  | 0.612 | 0.711 | 0.807 | 0.849 | 0.808 |
|  | Sailfish_cir | 0.556 | 0.526 | 0.531 | 0.513 | 0.507 | 0.516 | 0.515 |  | 0.646 | 0.589 | 0.516 | 0.407 | 0.307 |
| SCC | CircAST | 0.672 | 0.779 | 0.813 | 0.824 | 0.829 | 0.834 | 0.814 |  | 0.616 | 0.750 | 0.834 | 0.860 | 0.804 |
|  | Sailfish_cir | 0.737 | 0.718 | 0.717 | 0.709 | 0.705 | 0.711 | 0.710 |  | 0.732 | 0.725 | 0.711 | 0.673 | 0.625 |

*Note*: PCC, Pearson correlation coefficient; SCC, Spearman correlation coefficient.
