# Supplementary material for: CircAST: Full-length Assembly and Quantification of Alternatively Spliced Isoforms in Circular RNAs
Source: Genomics Proteomics Bioinformatics. 2020 Jan 31;17(5):522–34. doi: 10.1016/j.gpb.2019.03.004 (PMC7056934; doi:10.1016/j.gpb.2019.03.004)
Supplement: Supplementary Table S7 [file mmc7.docx]

**Table S7 Novel AS events in circular transcripts from Hs68 cells supported by ≥ 2 solid junction reads**

| **Chr** | **Location of**  **5' donor site** | **Location of**  **3' acceptor site** | **No. of forward splice junction reads** |
| --- | --- | --- | --- |
| Chr2 | 36,623,930 | 36,669,758 | 70 |
| Chr17 | 995,090 | 1,003,877 | 29 |
| Chr2 | 68,730,035 | 68,740,680 | 25 |
| Chr8 | 141,874,498 | 141,900,642 | 24 |
| Chr2 | 36,623,930 | 36,704,032 | 22 |
| Chr2 | 215,634,036 | 215,657,021 | 20 |
| Chr9 | 111,843,223 | 111,849,453 | 18 |
| Chr9 | 33,953,472 | 33,960,824 | 17 |
| Chr5 | 5,235,299 | 5,239,264 | 16 |
| Chr2 | 190,606,177 | 190,609,468 | 13 |
| Chr11 | 120,918,376 | 120,925,761 | 13 |
| Chr9 | 117,808,961 | 117,819,432 | 12 |
| Chr3 | 133,894,883 | 133,901,846 | 12 |
| Chr18 | 13,015,447 | 13,018,479 | 11 |
| Chr5 | 148,612,863 | 148,619,322 | 10 |
| Chr3 | 141,105,779 | 141,122,769 | 10 |
| Chr2 | 102,029,533 | 102,033,996 | 10 |
| Chr9 | 86,293,514 | 86,297,866 | 10 |
| Chr17 | 36,520,739 | 36,522,170 | 8 |
| Chr8 | 38,793,568 | 38,803,642 | 8 |
| Chr12 | 78,362,482 | 78,392,117 | 8 |
| Chr4 | 1,906,105 | 1,919,868 | 8 |
| Chr10 | 15,879,317 | 15,883,425 | 7 |
| Chr9 | 22,056,386 | 22,063,943 | 7 |
| Chr13 | 30,815,223 | 30,854,200 | 7 |
| Chr3 | 50,211,783 | 50,214,201 | 7 |
| Chr1 | 197,614,873 | 197,621,365 | 7 |
| Chr9 | 134,518,804 | 134,526,197 | 7 |
| Chr9 | 99,304,174 | 99,324,975 | 7 |
| Chr10 | 69,726,559 | 69,750,661 | 6 |
| Chr12 | 57,677,839 | 57,682,792 | 6 |
| Chr5 | 148,619,451 | 148,624,444 | 6 |
| Chr9 | 22,056,386 | 22,092,307 | 6 |
| Chr10 | 69,748,592 | 69,750,661 | 6 |
| Chr11 | 35,226,187 | 35,229,652 | 6 |
| Chr2 | 36,669,878 | 36,704,032 | 6 |
| Chr9 | 4,118,881 | 4,286,038 | 6 |
| Chr4 | 121,706,246 | 121,720,816 | 6 |
| Chr19 | 41,122,926 | 41,125,252 | 6 |
| Chr13 | 23,930,146 | 23,939,305 | 5 |
| Chr6 | 170,034,621 | 170,043,793 | 5 |
| Chr3 | 171,969,331 | 172,013,153 | 5 |
| Chr2 | 20,508,345 | 20,511,997 | 5 |
| Chr1 | 59,805,741 | 59,844,421 | 5 |
| Chr15 | 49,531,564 | 49,575,763 | 5 |
| Chr2 | 172,309,723 | 172,325,398 | 5 |
| Chr1 | 6,008,311 | 6,021,854 | 5 |
| Chr1 | 32,508,320 | 32,510,932 | 5 |
| Chr17 | 36,517,658 | 36,522,170 | 5 |
| Chr4 | 128,842,926 | 128,851,838 | 4 |
| Chr21 | 47,544,834 | 47,545,379 | 4 |
| Chr9 | 134,319,715 | 134,322,472 | 4 |
| Chr4 | 185,580,591 | 185,587,071 | 4 |
| Chr3 | 33,633,988 | 33,644,444 | 4 |
| Chr1 | 151,070,478 | 151,079,513 | 4 |
| Chr5 | 38,991,177 | 39,002,637 | 4 |
| Chr2 | 204,267,457 | 204,281,631 | 4 |
| Chr5 | 5,182,418 | 5,187,838 | 4 |
| Chr2 | 214,204,994 | 214,228,800 | 4 |
| Chr3 | 37,163,182 | 37,170,554 | 4 |
| Chr20 | 18,285,822 | 18,286,312 | 4 |
| Chr6 | 170,002,417 | 170,033,043 | 4 |
| Chr17 | 76,388,746 | 76,394,333 | 4 |
| Chr11 | 35,219,793 | 35,226,059 | 4 |
| Chr13 | 95,813,589 | 95,815,864 | 4 |
| Chr6 | 170,034,621 | 170,038,635 | 4 |
| Chr2 | 190,593,547 | 190,602,409 | 4 |
| Chr7 | 66,240,380 | 66,260,498 | 4 |
| Chr10 | 12,126,750 | 12,130,985 | 4 |
| Chr11 | 9,990,092 | 10,011,044 | 4 |
| Chr4 | 186,168,532 | 186,185,592 | 4 |
| Chr11 | 120,300,226 | 120,302,480 | 3 |
| Chr10 | 105,768,114 | 105,777,918 | 3 |
| Chr7 | 140,268,613 | 140,273,608 | 3 |
| Chr11 | 126,277,529 | 126,278,202 | 3 |
| Chr11 | 85,685,855 | 85,692,172 | 3 |
| Chr3 | 66,457,915 | 66,463,295 | 3 |
| Chr2 | 173,431,661 | 173,435,454 | 3 |
| Chr17 | 35,800,763 | 35,804,798 | 3 |
| Chr2 | 32,626,453 | 32,631,567 | 3 |
| Chr18 | 56,606,853 | 56,620,750 | 3 |
| Chr7 | 131,099,469 | 131,122,544 | 3 |
| Chr1 | 51,869,204 | 51,873,807 | 3 |
| Chr10 | 103,552,700 | 103,557,737 | 3 |
| Chr1 | 6,022,009 | 6,029,147 | 3 |
| Chr7 | 44,005,994 | 44,012,229 | 3 |
| Chr8 | 145,245,838 | 145,247,212 | 3 |
| Chr17 | 57,140,009 | 57,148,184 | 3 |
| Chr8 | 103,358,623 | 103,372,299 | 3 |
| Chr2 | 36,623,930 | 36,726,362 | 3 |
| Chr9 | 111,849,622 | 111,855,755 | 3 |
| Chr17 | 19,843,162 | 19,845,139 | 3 |
| Chr8 | 141,829,119 | 141,856,359 | 3 |
| Chr12 | 129,294,018 | 129,299,320 | 3 |
| Chr22 | 38,934,617 | 38,948,671 | 3 |
| Chr7 | 121,753,774 | 121,756,687 | 3 |
| Chr2 | 62,100,428 | 62,104,055 | 3 |
| Chr17 | 73,238,992 | 73,239,528 | 3 |
| Chr10 | 12,131,254 | 12,136,072 | 3 |
| Chr14 | 35,074,923 | 35,078,844 | 3 |
| Chr15 | 41,657,787 | 41,667,910 | 3 |
| Chr19 | 13,186,485 | 13,192,494 | 3 |
| Chr6 | 42,585,245 | 42,600,290 | 3 |
| Chr12 | 101,365,184 | 101,381,317 | 3 |
| Chr15 | 49,531,564 | 49,584,524 | 3 |
| Chr19 | 41,120,352 | 41,123,006 | 3 |
| Chr2 | 101,644,894 | 101,650,006 | 3 |
| Chr9 | 111,800,397 | 111,819,471 | 2 |
| Chr1 | 233,344,435 | 233,353,777 | 2 |
| Chr8 | 141,810,675 | 141,828,376 | 2 |
| Chr9 | 99,296,824 | 99,324,975 | 2 |
| Chr2 | 200,213,896 | 200,298,061 | 2 |
| Chr14 | 37,754,652 | 37,838,722 | 2 |
| Chr9 | 96,259,881 | 96,277,949 | 2 |
| Chr1 | 240,497,529 | 240,555,811 | 2 |
| Chr2 | 45,774,751 | 45,780,762 | 2 |
| Chr10 | 99,220,764 | 99,221,585 | 2 |
| Chr12 | 51,447,643 | 51,450,133 | 2 |
| Chr9 | 22,064,017 | 22,066,234 | 2 |
| Chr15 | 76,165,909 | 76,171,439 | 2 |
| Chr14 | 78,023,489 | 78,036,727 | 2 |
| Chr11 | 46,515,754 | 46,534,277 | 2 |
| Chr7 | 65,444,528 | 65,445,211 | 2 |
| Chr3 | 56,697,600 | 56,702,425 | 2 |
| Chr2 | 29,366,811 | 29,375,551 | 2 |
| Chr19 | 45,645,716 | 45,648,112 | 2 |
| Chr10 | 12,129,728 | 12,139,683 | 2 |
| Chr22 | 24,434,909 | 24,439,366 | 2 |
| Chr19 | 5,016,350 | 5,039,847 | 2 |
| Chr17 | 79,244,824 | 79,249,769 | 2 |
| Chr5 | 64,520,227 | 64,521,913 | 2 |
| Chr12 | 66,603,981 | 66,610,951 | 2 |
| Chr10 | 12,123,626 | 12,129,534 | 2 |
| Chr16 | 12,146,079 | 12,162,914 | 2 |
| Chr1 | 229,596,516 | 229,600,371 | 2 |
| Chr17 | 57,812,834 | 57,816,198 | 2 |
| Chr22 | 46,114,373 | 46,134,611 | 2 |
| Chr1 | 53,326,543 | 53,332,175 | 2 |
| Chr9 | 22,065,756 | 22,097,257 | 2 |
| Chr15 | 50,593,565 | 50,596,163 | 2 |
| Chr16 | 521,389 | 538,851 | 2 |
| Chr7 | 23,381,808 | 23,385,559 | 2 |
| Chr6 | 159,026,379 | 159,029,365 | 2 |
| Chr19 | 34,949,829 | 34,954,931 | 2 |
| Chr11 | 3,756,554 | 3,774,546 | 2 |
| Chr15 | 66,021,585 | 66,023,980 | 2 |
| Chr14 | 71,514,701 | 71,522,221 | 2 |
| Chr4 | 1,936,989 | 1,941,381 | 2 |
| Chr12 | 2,613,705 | 2,614,008 | 2 |
| Chr1 | 42,776,781 | 42,789,355 | 2 |
| Chr15 | 30,058,745 | 30,092,849 | 2 |
| Chr6 | 38,029,551 | 38,084,348 | 2 |
| Chr17 | 66,352,945 | 66,381,205 | 2 |
| Chr3 | 123,359,351 | 123,367,818 | 2 |
| Chr3 | 12,645,788 | 12,650,265 | 2 |
| Chr15 | 76,584,854 | 76,587,932 | 2 |
| Chr18 | 76,967,012 | 77,013,381 | 2 |
| Chr10 | 1,118,233 | 1,125,951 | 2 |
| Chr5 | 78,945,013 | 78,964,715 | 2 |
| Chr20 | 32,661,672 | 32,664,508 | 2 |
| Chr6 | 144,086,935 | 144,095,196 | 2 |
| Chr12 | 51,442,968 | 51,450,133 | 2 |
| Chr10 | 70,723,225 | 70,726,774 | 2 |
| Chr17 | 36,936,857 | 36,943,076 | 2 |
| Chr10 | 96,256,929 | 96,267,024 | 2 |
| Chr1 | 36,297,786 | 36,299,591 | 2 |
| Chr12 | 64,015,117 | 64,020,249 | 2 |
| Chr1 | 70,766,591 | 70,779,428 | 2 |
| Chr11 | 68,341,692 | 68,350,511 | 2 |
| Chr5 | 5,262,896 | 5,303,685 | 2 |
| Chr5 | 33,549,488 | 33,576,159 | 2 |
| Chr13 | 77,799,689 | 77,817,194 | 2 |
| Chr15 | 63,848,947 | 63,852,056 | 2 |
| Chr14 | 76,644,385 | 76,662,221 | 2 |
| Chr11 | 35,219,793 | 35,229,652 | 2 |
| Chr2 | 190,593,547 | 190,606,068 | 2 |
| Chr4 | 185,580,591 | 185,593,327 | 2 |
| Chr12 | 64,015,117 | 64,038,183 | 2 |
| Chr12 | 110,824,274 | 110,826,317 | 2 |
| Chr2 | 203,978,041 | 203,986,977 | 2 |
| Chr6 | 31,616,528 | 31,616,976 | 2 |
| Chr10 | 93,588,163 | 93,593,610 | 2 |
| Chr8 | 1,824,900 | 1,830,801 | 2 |
| Chr5 | 80,390,811 | 80,409,357 | 2 |
| Chr19 | 41,120,352 | 41,125,252 | 2 |
| Chr7 | 23,015,924 | 23,016,960 | 2 |
| Chr11 | 85,733,512 | 85,742,511 | 2 |
| Chr9 | 88,284,493 | 88,292,351 | 2 |
| Chr5 | 145,144,563 | 145,197,457 | 2 |
| Chr17 | 982,630 | 994,905 | 2 |
| Chr10 | 12,139,995 | 12,143,041 | 2 |
| Chr18 | 19,378,189 | 19,383,868 | 2 |
| Chr17 | 66,429,716 | 66,431,761 | 2 |
| Chr13 | 100,909,927 | 100,920,943 | 2 |
| Chr4 | 151,727,556 | 151,738,276 | 2 |
| Chr5 | 233,760 | 236,543 | 2 |
| Chr18 | 76,886,375 | 76,914,504 | 2 |
| Chr3 | 111,664,204 | 111,672,777 | 2 |
| Chr3 | 119,222,868 | 119,236,052 | 2 |
