# Supplementary material for: CircAST: Full-length Assembly and Quantification of Alternatively Spliced Isoforms in Circular RNAs
Source: Genomics Proteomics Bioinformatics. 2020 Jan 31;17(5):522–34. doi: 10.1016/j.gpb.2019.03.004 (PMC7056934; doi:10.1016/j.gpb.2019.03.004)
Supplement: Supplementary Table S8 [file mmc8.docx]

**Table S8 Novel AS events in circular transcripts from HEK293 cells supported by ≥ 2 solid junction reads**

| **Chr** | **Location of**  **5' donor site** | **Location of**  **3' acceptor site** | **No. of forward splice junction reads** |
| --- | --- | --- | --- |
| Chr9 | 33,953,472 | 33,960,824 | 120 |
| Chr14 | 105,911,848 | 105,916,395 | 29 |
| Chr1 | 225,142,800 | 225,152,181 | 26 |
| Chr13 | 78,293,806 | 78,317,151 | 23 |
| Chr1 | 235,647,831 | 235,657,991 | 23 |
| Chr8 | 1,824,900 | 1,830,801 | 22 |
| Chr2 | 215,634,036 | 215,657,021 | 18 |
| Chr9 | 111,843,223 | 111,849,453 | 17 |
| Chr22 | 42,206,004 | 42,209,268 | 16 |
| Chr10 | 105,768,114 | 105,777,918 | 15 |
| Chr1 | 6,012,896 | 6,029,147 | 15 |
| Chr9 | 134,319,715 | 134,322,472 | 15 |
| Chr19 | 55,853,414 | 55,854,110 | 14 |
| Chr10 | 12,126,750 | 12,130,985 | 13 |
| Chr17 | 19,843,162 | 19,845,139 | 12 |
| Chr12 | 51,447,643 | 51,450,133 | 12 |
| Chr10 | 12,131,254 | 12,136,072 | 12 |
| Chr12 | 111,951,343 | 111,956,053 | 11 |
| Chr1 | 42,776,781 | 42,789,355 | 11 |
| Chr1 | 21,076,375 | 21,091,870 | 10 |
| Chr20 | 47,700,699 | 47,705,784 | 10 |
| Chr9 | 97,686,457 | 97,717,459 | 10 |
| Chr11 | 22,232,860 | 22,242,643 | 10 |
| Chr10 | 12,139,995 | 12,143,041 | 10 |
| Chr17 | 36,517,658 | 36,522,170 | 10 |
| Chr12 | 51,442,968 | 51,449,618 | 10 |
| Chr3 | 47,467,659 | 47,476,498 | 10 |
| Chr16 | 47,533,805 | 47,545,576 | 9 |
| Chr3 | 133,894,883 | 133,901,846 | 9 |
| Chr18 | 13,015,447 | 13,018,479 | 8 |
| Chr17 | 36,520,739 | 36,522,170 | 8 |
| Chr2 | 68,730,035 | 68,740,680 | 8 |
| Chr3 | 52,780,920 | 52,785,948 | 8 |
| Chr2 | 136,505,939 | 136,513,087 | 8 |
| Chr5 | 151,166,276 | 151,170,450 | 8 |
| Chr9 | 134,322,593 | 134,330,463 | 7 |
| Chr12 | 53,416,411 | 53,421,799 | 7 |
| Chr9 | 96,259,881 | 96,277,949 | 7 |
| ChrX | 73,051,109 | 73,057,275 | 6 |
| Chr1 | 146,747,921 | 146,756,024 | 6 |
| Chr19 | 5,047,680 | 5,077,378 | 6 |
| Chr8 | 141,874,498 | 141,900,642 | 6 |
| Chr2 | 242,606,253 | 242,607,955 | 6 |
| Chr8 | 124,089,497 | 124,096,402 | 6 |
| Chr7 | 72,880,731 | 72,884,675 | 6 |
| Chr3 | 33,633,988 | 33,644,444 | 6 |
| Chr11 | 68,115,711 | 68,131,215 | 6 |
| Chr10 | 12,155,063 | 12,160,748 | 6 |
| Chr1 | 233,353,930 | 233,372,591 | 6 |
| Chr1 | 31,447,649 | 31,454,159 | 6 |
| Chr1 | 1,275,029 | 1,275,418 | 6 |
| Chr17 | 35,800,763 | 35,804,798 | 6 |
| Chr3 | 196,817,897 | 196,842,798 | 5 |
| Chr16 | 75,651,170 | 75,654,164 | 5 |
| Chr16 | 70,572,363 | 70,575,572 | 5 |
| Chr1 | 155,743,001 | 155,746,186 | 5 |
| Chr2 | 10,784,498 | 10,797,868 | 5 |
| Chr1 | 6,022,009 | 6,029,147 | 5 |
| Chr20 | 35,695,524 | 35,696,389 | 5 |
| Chr9 | 138,742,307 | 138,758,302 | 5 |
| Chr15 | 101,970,268 | 101,972,195 | 4 |
| Chr1 | 28,599,304 | 28,600,551 | 4 |
| ChrX | 102,082,072 | 102,100,785 | 4 |
| Chr13 | 28,752,072 | 28,794,368 | 4 |
| Chr15 | 101,933,629 | 101,968,097 | 4 |
| Chr1 | 151,060,773 | 151,065,668 | 4 |
| Chr11 | 76,169,402 | 76,174,865 | 4 |
| Chr10 | 15,879,317 | 15,883,425 | 4 |
| Chr16 | 21,976,826 | 21,982,846 | 4 |
| Chr10 | 103,552,700 | 103,557,737 | 4 |
| Chr1 | 46,531,851 | 46,543,187 | 4 |
| Chr1 | 35,853,205 | 35,855,549 | 4 |
| Chr11 | 85,733,512 | 85,742,511 | 4 |
| Chr10 | 116,919,975 | 116,930,795 | 4 |
| Chr4 | 887,797 | 891,821 | 4 |
| Chr5 | 145,144,563 | 145,197,457 | 4 |
| Chr1 | 160,194,339 | 160,195,381 | 4 |
| Chr9 | 6,421,142 | 6,460,573 | 4 |
| Chr9 | 88,201,875 | 88,204,443 | 4 |
| Chr22 | 42,206,295 | 42,209,755 | 4 |
| Chr2 | 36,669,878 | 36,704,032 | 4 |
| Chr17 | 80,521,424 | 80,529,600 | 4 |
| Chr1 | 202,409,916 | 202,418,117 | 4 |
| Chr16 | 71,692,718 | 71,701,081 | 4 |
| Chr6 | 170,034,621 | 170,038,635 | 4 |
| Chr1 | 197,576,304 | 197,586,789 | 4 |
| Chr5 | 122,435,656 | 122,506,460 | 4 |
| Chr1 | 160,210,160 | 160,231,075 | 4 |
| Chr5 | 176,370,489 | 176,382,960 | 4 |
| Chr12 | 50,829,407 | 50,834,222 | 4 |
| Chr5 | 170,343,588 | 170,346,445 | 4 |
| Chr17 | 79,244,824 | 79,249,769 | 4 |
| Chr5 | 171,297,862 | 171,303,289 | 4 |
| Chr4 | 128,843,118 | 128,854,140 | 3 |
| Chr16 | 11,990,642 | 11,991,851 | 3 |
| Chr7 | 35,184,702 | 35,189,700 | 3 |
| Chr7 | 151,921,264 | 151,927,008 | 3 |
| Chr17 | 60,107,012 | 60,108,805 | 3 |
| Chr1 | 35,847,034 | 35,851,043 | 3 |
| Chr3 | 142,467,302 | 142,499,676 | 3 |
| Chr1 | 32,508,320 | 32,510,932 | 3 |
| Chr20 | 47,700,699 | 47,704,546 | 3 |
| Chr7 | 140,494,267 | 140,501,212 | 3 |
| Chr16 | 87,788,898 | 87,795,555 | 3 |
| Chr7 | 138,210,102 | 138,223,402 | 3 |
| Chr5 | 74,130,422 | 74,135,899 | 3 |
| Chr4 | 20,525,800 | 20,530,572 | 3 |
| Chr4 | 154,214,285 | 154,216,465 | 3 |
| Chr1 | 28,595,759 | 28,598,795 | 3 |
| Chr4 | 128,842,926 | 128,851,838 | 3 |
| Chr1 | 243,736,350 | 243,800,913 | 3 |
| Chr21 | 44,437,117 | 44,441,413 | 3 |
| Chr3 | 119,219,707 | 119,222,801 | 3 |
| Chr1 | 878,438 | 879,078 | 3 |
| Chr13 | 51,504,895 | 51,517,457 | 3 |
| Chr19 | 34,945,258 | 34,949,674 | 3 |
| Chr11 | 85,685,855 | 85,692,172 | 3 |
| Chr17 | 28,598,406 | 28,601,060 | 3 |
| Chr18 | 19,383,975 | 19,399,456 | 3 |
| Chr1 | 70,766,591 | 70,779,428 | 3 |
| Chr1 | 65,095,164 | 65,099,727 | 3 |
| Chr9 | 125,618,157 | 125,620,948 | 3 |
| Chr5 | 109,051,965 | 109,091,030 | 3 |
| Chr4 | 128,854,248 | 128,861,008 | 3 |
| Chr21 | 44,279,832 | 44,283,550 | 2 |
| Chr1 | 41,512,270 | 41,536,267 | 2 |
| Chr15 | 41,657,787 | 41,669,394 | 2 |
| Chr9 | 111,849,622 | 111,855,755 | 2 |
| Chr4 | 83,795,904 | 83,799,883 | 2 |
| Chr2 | 242,594,062 | 242,606,060 | 2 |
| Chr4 | 48,380,077 | 48,384,585 | 2 |
| Chr2 | 204,259,569 | 204,267,299 | 2 |
| Chr17 | 37,866,134 | 37,866,593 | 2 |
| Chr1 | 6,008,311 | 6,021,854 | 2 |
| Chr17 | 44,145,033 | 44,171,926 | 2 |
| Chr6 | 159,026,379 | 159,029,365 | 2 |
| Chr17 | 11,984,847 | 12,013,692 | 2 |
| Chr10 | 12,042,008 | 12,046,529 | 2 |
| Chr3 | 56,703,819 | 56,705,628 | 2 |
| Chr21 | 34,614,282 | 34,617,256 | 2 |
| Chr7 | 140,482,957 | 140,494,108 | 2 |
| Chr3 | 47,468,752 | 47,470,003 | 2 |
| Chr12 | 51,445,990 | 51,450,133 | 2 |
| Chr6 | 170,034,621 | 170,043,793 | 2 |
| Chr5 | 179,135,381 | 179,136,874 | 2 |
| Chr4 | 151,682,999 | 151,727,423 | 2 |
| Chr21 | 37,775,149 | 37,781,672 | 2 |
| Chr5 | 179,251,323 | 179,260,587 | 2 |
| Chr15 | 41,657,787 | 41,667,910 | 2 |
| Chr20 | 33,935,075 | 33,969,721 | 2 |
| Chr5 | 179,133,332 | 179,135,240 | 2 |
