# Supplementary material for: CircAST: Full-length Assembly and Quantification of Alternatively Spliced Isoforms in Circular RNAs
Source: Genomics Proteomics Bioinformatics. 2020 Jan 31;17(5):522–34. doi: 10.1016/j.gpb.2019.03.004 (PMC7056934; doi:10.1016/j.gpb.2019.03.004)
Supplement: Supplementary Table S9 [file mmc9.docx]

**Table S9 Novel AS events in circular transcripts from HeLa cells supported by ≥ 2 solid junction reads**

| **Chr** | **Location of**  **5' donor site** | **Location of**  **3' acceptor site** | **No. of forward splice junction reads** |
| --- | --- | --- | --- |
| Chr19 | 34,699,956 | 34,706,501 | 157 |
| Chr11 | 85,685,855 | 85,692,172 | 41 |
| Chr7 | 11,101,712 | 11,150,977 | 38 |
| Chr9 | 134,518,804 | 134,526,197 | 37 |
| Chr8 | 1,824,900 | 1,830,801 | 37 |
| Chr14 | 97,321,693 | 97,322,865 | 36 |
| Chr9 | 133,333,976 | 133,342,112 | 30 |
| Chr8 | 141,874,498 | 141,900,642 | 29 |
| Chr9 | 134,319,715 | 134,322,472 | 27 |
| Chr5 | 620,376 | 633,884 | 25 |
| Chr1 | 225,142,800 | 225,152,181 | 24 |
| Chr10 | 15,879,317 | 15,883,425 | 21 |
| Chr13 | 111,857,720 | 111,870,026 | 21 |
| ChrX | 150,791,536 | 150,817,087 | 20 |
| Chr9 | 133,355,836 | 133,370,254 | 20 |
| Chr7 | 5,024,706 | 5,028,694 | 19 |
| Chr12 | 5,841,796 | 5,848,483 | 19 |
| Chr3 | 133,894,883 | 133,901,846 | 17 |
| Chr10 | 70,097,090 | 70,098,260 | 17 |
| Chr9 | 111,843,223 | 111,849,453 | 16 |
| Chr17 | 36,517,658 | 36,522,170 | 16 |
| Chr20 | 57,243,183 | 57,245,568 | 15 |
| Chr5 | 78,945,013 | 78,964,715 | 14 |
| Chr12 | 51,442,968 | 51,450,133 | 14 |
| Chr1 | 197,614,873 | 197,621,365 | 14 |
| Chr3 | 33,633,988 | 33,644,444 | 13 |
| Chr9 | 134,322,593 | 134,330,463 | 12 |
| Chr18 | 13,030,607 | 13,040,829 | 12 |
| Chr3 | 141,105,779 | 141,122,769 | 12 |
| Chr1 | 235,647,831 | 235,657,991 | 12 |
| Chr1 | 225,161,855 | 225,195,115 | 12 |
| Chr7 | 23,381,808 | 23,385,559 | 12 |
| Chr18 | 19,378,189 | 19,383,868 | 12 |
| Chr12 | 51,447,643 | 51,450,133 | 11 |
| Chr2 | 36,739,537 | 36,744,470 | 11 |
| Chr15 | 101,970,268 | 101,972,195 | 11 |
| Chr12 | 117,383,333 | 117,402,502 | 11 |
| Chr16 | 75,637,069 | 75,646,141 | 10 |
| Chr5 | 624,694 | 635,487 | 10 |
| Chr9 | 88,201,875 | 88,204,443 | 10 |
| Chr7 | 26,232,993 | 26,235,467 | 10 |
| Chr2 | 215,634,036 | 215,657,021 | 10 |
| Chr17 | 80,858,607 | 80,863,812 | 10 |
| Chr11 | 120,918,376 | 120,925,761 | 9 |
| Chr5 | 179,135,381 | 179,136,874 | 9 |
| Chr18 | 13,015,447 | 13,018,479 | 9 |
| Chr9 | 33,953,472 | 33,960,824 | 9 |
| Chr11 | 85,707,972 | 85,712,078 | 9 |
| Chr9 | 111,849,622 | 111,855,755 | 9 |
| Chr9 | 86,293,514 | 86,297,866 | 9 |
| Chr1 | 234,596,142 | 234,601,402 | 9 |
| Chr7 | 72,880,731 | 72,884,675 | 8 |
| Chr1 | 225,155,285 | 225,161,793 | 8 |
| Chr9 | 22,056,386 | 22,061,952 | 8 |
| Chr5 | 37,162,668 | 37,165,641 | 8 |
| Chr4 | 1,936,989 | 1,952,799 | 8 |
| Chr11 | 85,733,512 | 85,742,511 | 8 |
| Chr3 | 5,249,948 | 5,252,805 | 8 |
| Chr10 | 123,658,484 | 123,659,381 | 7 |
| Chr10 | 69,726,559 | 69,750,661 | 7 |
| Chr5 | 40,767,792 | 40,771,821 | 7 |
| Chr1 | 58,993,007 | 58,999,625 | 7 |
| Chr19 | 3,546,166 | 3,547,270 | 7 |
| Chr13 | 114,175,048 | 114,193,672 | 7 |
| Chr15 | 76,584,854 | 76,587,932 | 7 |
| Chr1 | 225,267,250 | 225,270,251 | 7 |
| Chr3 | 124,536,557 | 124,540,160 | 7 |
| Chr15 | 41,657,787 | 41,667,910 | 7 |
| Chr16 | 3,726,146 | 3,729,720 | 7 |
| Chr2 | 32,399,216 | 32,409,342 | 7 |
| Chr13 | 95,813,589 | 95,815,864 | 7 |
| Chr9 | 138,742,307 | 138,758,302 | 6 |
| Chr3 | 132,400,934 | 132,402,243 | 6 |
| Chr15 | 101,933,629 | 101,968,097 | 6 |
| Chr9 | 88,292,497 | 88,296,183 | 6 |
| Chr22 | 46,098,727 | 46,125,305 | 6 |
| Chr5 | 170,308,900 | 170,336,665 | 6 |
| Chr5 | 151,166,276 | 151,170,450 | 6 |
| Chr1 | 225,156,576 | 225,195,115 | 6 |
| Chr11 | 47,510,576 | 47,522,413 | 6 |
| Chr19 | 1,422,395 | 1,428,841 | 6 |
| Chr1 | 225,373,127 | 225,391,885 | 6 |
| Chr10 | 70,204,840 | 70,209,785 | 6 |
| Chr13 | 96,638,686 | 96,648,317 | 6 |
| Chr10 | 35,805,551 | 35,818,901 | 6 |
| Chr9 | 96,259,881 | 96,277,949 | 6 |
| Chr1 | 225,156,576 | 225,190,485 | 6 |
| Chr12 | 110,825,697 | 110,832,906 | 5 |
| Chr8 | 101,725,017 | 101,727,690 | 5 |
| Chr9 | 133,352,348 | 133,355,772 | 5 |
| Chr17 | 57,812,834 | 57,816,198 | 5 |
| Chr5 | 73,144,912 | 73,153,481 | 5 |
| Chr1 | 6,022,009 | 6,029,147 | 5 |
| Chr6 | 42,585,245 | 42,600,290 | 5 |
| Chr1 | 225,373,127 | 225,418,775 | 5 |
| Chr18 | 76,914,555 | 76,953,183 | 5 |
| Chr5 | 72,354,351 | 72,364,477 | 5 |
| Chr11 | 126,278,089 | 126,279,163 | 5 |
| Chr5 | 637,933 | 640,523 | 5 |
| ChrX | 150,773,206 | 150,789,402 | 5 |
| Chr10 | 12,131,254 | 12,136,072 | 5 |
| Chr11 | 57,188,537 | 57,191,455 | 5 |
| Chr12 | 51,442,968 | 51,449,618 | 5 |
| Chr8 | 101,724,685 | 101,725,315 | 5 |
| Chr2 | 36,669,878 | 36,704,032 | 5 |
| Chr13 | 96,624,925 | 96,638,587 | 5 |
| Chr1 | 70,761,944 | 70,779,428 | 4 |
| Chr2 | 239,090,827 | 239,092,661 | 4 |
| Chr3 | 56,697,600 | 56,702,425 | 4 |
| Chr9 | 22,056,386 | 22,063,943 | 4 |
| Chr12 | 124,934,413 | 124,950,719 | 4 |
| Chr1 | 197,621,445 | 197,641,167 | 4 |
| Chr1 | 151,070,478 | 151,079,513 | 4 |
| Chr5 | 38,991,177 | 39,002,637 | 4 |
| Chr11 | 57,191,501 | 57,193,462 | 4 |
| Chr19 | 5,033,042 | 5,047,487 | 4 |
| Chr20 | 57,242,653 | 57,245,568 | 4 |
| Chr2 | 36,623,930 | 36,669,758 | 4 |
| Chr21 | 27,347,541 | 27,354,657 | 4 |
| Chr5 | 179,250,053 | 179,251,182 | 4 |
| Chr17 | 80,851,508 | 80,863,812 | 4 |
| Chr13 | 111,319,820 | 111,335,398 | 4 |
| Chr2 | 44,139,697 | 44,145,395 | 4 |
| Chr1 | 155,365,344 | 155,408,118 | 4 |
| Chr6 | 159,002,027 | 159,006,336 | 4 |
| Chr5 | 43,297,268 | 43,298,620 | 4 |
| Chr10 | 69,726,559 | 69,749,968 | 4 |
| Chr4 | 186,168,532 | 186,185,592 | 4 |
| Chr22 | 29,095,925 | 29,105,994 | 4 |
| Chr19 | 3,548,028 | 3,550,982 | 4 |
| Chr11 | 57,182,579 | 57,185,221 | 3 |
| Chr5 | 56,155,742 | 56,161,167 | 3 |
| Chr2 | 190,656,667 | 190,670,378 | 3 |
| Chr1 | 151,633,338 | 151,640,948 | 3 |
| Chr21 | 38,463,713 | 38,467,650 | 3 |
| Chr10 | 12,042,008 | 12,046,529 | 3 |
| Chr11 | 46,515,754 | 46,534,277 | 3 |
| Chr11 | 46,565,591 | 46,568,663 | 3 |
| Chr16 | 70,572,363 | 70,578,341 | 3 |
| Chr1 | 234,561,539 | 234,563,322 | 3 |
| Chr10 | 70,097,090 | 70,098,897 | 3 |
| Chr17 | 80,767,658 | 80,828,100 | 3 |
| Chr1 | 233,353,930 | 233,372,591 | 3 |
| Chr12 | 50,393,054 | 50,394,955 | 3 |
| Chr16 | 70,572,363 | 70,575,572 | 3 |
| Chr9 | 22,056,386 | 22,092,307 | 3 |
| Chr21 | 17,138,460 | 17,163,821 | 3 |
| Chr8 | 145,245,838 | 145,247,212 | 3 |
| Chr11 | 66,136,142 | 66,136,840 | 3 |
| Chr5 | 179,251,323 | 179,260,032 | 3 |
| Chr15 | 76,165,909 | 76,170,302 | 3 |
| Chr17 | 20,163,607 | 20,209,336 | 3 |
| Chr8 | 104,415,552 | 104,419,862 | 3 |
| Chr8 | 141,829,119 | 141,856,359 | 3 |
| Chr12 | 51,445,990 | 51,450,133 | 3 |
| Chr17 | 80,521,424 | 80,529,600 | 3 |
| Chr18 | 9,122,679 | 9,126,829 | 3 |
| Chr17 | 74,283,978 | 74,287,096 | 3 |
| Chr12 | 110,824,274 | 110,832,906 | 3 |
| Chr12 | 30,814,200 | 30,816,423 | 3 |
| Chr18 | 19,348,713 | 19,358,064 | 3 |
| Chr8 | 37,972,518 | 37,976,788 | 3 |
| Chr1 | 6,008,311 | 6,021,854 | 3 |
| Chr16 | 88,052,273 | 88,066,715 | 3 |
| Chr5 | 179,134,191 | 179,136,874 | 3 |
| Chr3 | 195,606,046 | 195,610,028 | 3 |
| Chr11 | 46,515,754 | 46,563,495 | 3 |
| Chr17 | 74,286,158 | 74,300,496 | 3 |
| Chr9 | 139,794,972 | 139,804,372 | 3 |
| Chr7 | 24,681,487 | 24,703,209 | 3 |
| Chr5 | 109,155,588 | 109,159,424 | 3 |
| Chr11 | 85,693,046 | 85,707,869 | 3 |
| Chr12 | 50,393,516 | 50,396,030 | 3 |
| Chr3 | 169,854,453 | 169,889,161 | 3 |
| Chr16 | 47,533,805 | 47,545,576 | 3 |
| Chr20 | 47,588,962 | 47,591,303 | 3 |
| Chr4 | 121,706,246 | 121,720,816 | 3 |
| Chr7 | 157,016,045 | 157,023,774 | 3 |
| Chr3 | 56,703,819 | 56,705,628 | 3 |
| Chr10 | 12,139,995 | 12,143,041 | 3 |
| Chr17 | 17,077,389 | 17,079,740 | 3 |
| Chr13 | 95,818,621 | 95,829,961 | 3 |
| Chr5 | 109,124,749 | 109,152,974 | 2 |
| Chr2 | 69,297,860 | 69,300,154 | 2 |
| Chr1 | 234,569,317 | 234,582,549 | 2 |
| Chr12 | 50,856,413 | 50,867,198 | 2 |
| Chr1 | 44,778,900 | 44,804,717 | 2 |
| Chr9 | 133,329,760 | 133,339,498 | 2 |
| Chr18 | 13,037,300 | 13,040,829 | 2 |
| Chr20 | 17,929,620 | 17,930,776 | 2 |
| Chr19 | 12,825,983 | 12,826,219 | 2 |
| Chr17 | 76,395,618 | 76,399,649 | 2 |
| Chr9 | 99,286,007 | 99,301,360 | 2 |
| Chr2 | 101,898,519 | 101,905,435 | 2 |
| Chr12 | 110,824,274 | 110,826,317 | 2 |
| Chr9 | 114,842,445 | 114,864,456 | 2 |
| Chr5 | 50,055,566 | 50,057,655 | 2 |
| Chr9 | 115,171,301 | 115,181,140 | 2 |
| Chr17 | 80,851,508 | 80,861,281 | 2 |
| Chr9 | 125,751,750 | 125,758,320 | 2 |
| Chr1 | 53,326,543 | 53,332,175 | 2 |
| Chr15 | 93,545,547 | 93,552,375 | 2 |
| Chr2 | 32,658,872 | 32,661,121 | 2 |
| Chr17 | 37,866,134 | 37,866,593 | 2 |
| Chr2 | 177,162,623 | 177,191,553 | 2 |
| Chr18 | 13,038,578 | 13,042,203 | 2 |
| Chr5 | 50,090,166 | 50,092,816 | 2 |
| Chr16 | 89,961,545 | 89,964,965 | 2 |
| Chr2 | 61,715,406 | 61,717,777 | 2 |
| Chr15 | 72,302,788 | 72,313,259 | 2 |
| Chr3 | 119,219,707 | 119,232,488 | 2 |
| Chr11 | 3,756,554 | 3,774,546 | 2 |
| Chr5 | 14,751,348 | 14,758,589 | 2 |
| Chr7 | 26,232,993 | 26,236,021 | 2 |
| Chr13 | 77,799,689 | 77,817,194 | 2 |
| Chr2 | 106,746,234 | 106,774,514 | 2 |
| Chr20 | 47,589,821 | 47,592,553 | 2 |
| Chr2 | 233,613,792 | 233,625,190 | 2 |
| Chr15 | 101,929,769 | 101,938,609 | 2 |
| Chr3 | 27,475,595 | 27,478,879 | 2 |
| Chr12 | 129,294,018 | 129,299,320 | 2 |
| Chr17 | 66,352,945 | 66,381,205 | 2 |
| Chr20 | 23,375,636 | 23,377,709 | 2 |
| Chr2 | 172,803,303 | 172,821,859 | 2 |
| Chr7 | 105,103,197 | 105,108,784 | 2 |
| Chr19 | 5,047,680 | 5,077,378 | 2 |
| Chr17 | 19,843,162 | 19,845,139 | 2 |
| Chr17 | 80,915,370 | 80,962,991 | 2 |
| Chr18 | 76,967,012 | 77,013,381 | 2 |
| Chr10 | 1,118,233 | 1,125,951 | 2 |
| Chr1 | 236,976,144 | 236,979,749 | 2 |
| Chr20 | 50,286,648 | 50,290,692 | 2 |
| Chr10 | 1,123,943 | 1,130,343 | 2 |
| Chr16 | 56,419,934 | 56,435,645 | 2 |
| Chr11 | 78,180,359 | 78,204,109 | 2 |
| Chr3 | 149,613,347 | 149,629,765 | 2 |
| Chr17 | 76,388,746 | 76,394,333 | 2 |
| Chr21 | 44,437,117 | 44,441,413 | 2 |
| Chr9 | 22,064,017 | 22,096,371 | 2 |
| Chr19 | 34,687,668 | 34,706,029 | 2 |
| Chr6 | 99,913,012 | 99,916,413 | 2 |
| Chr3 | 136,708,407 | 136,714,256 | 2 |
| Chr1 | 156,713,670 | 156,714,800 | 2 |
| ChrX | 67,733,247 | 67,741,214 | 2 |
| Chr17 | 80,842,078 | 80,858,527 | 2 |
| Chr4 | 77,055,515 | 77,065,302 | 2 |
| Chr18 | 13,030,607 | 13,038,369 | 2 |
| Chr12 | 123,058,927 | 123,060,347 | 2 |
| Chr10 | 12,133,683 | 12,139,683 | 2 |
| Chr11 | 18,017,456 | 18,028,138 | 2 |
| Chr17 | 17,076,129 | 17,079,740 | 2 |
| Chr20 | 32,878,255 | 32,879,225 | 2 |
| Chr11 | 126,277,244 | 126,278,202 | 2 |
| Chr15 | 41,650,456 | 41,663,725 | 2 |
| Chr1 | 109,954,806 | 109,957,859 | 2 |
| Chr15 | 59,502,799 | 59,510,090 | 2 |
| Chr10 | 103,384,567 | 103,432,672 | 2 |
| Chr5 | 93,987,550 | 93,990,335 | 2 |
