# Supplementary material for: CircAST: Full-length Assembly and Quantification of Alternatively Spliced Isoforms in Circular RNAs
Source: Genomics Proteomics Bioinformatics. 2020 Jan 31;17(5):522–34. doi: 10.1016/j.gpb.2019.03.004 (PMC7056934; doi:10.1016/j.gpb.2019.03.004)
Supplement: Supplementary Table S10 [file mmc10.docx]

**Table S10 Novel AS events in circular transcripts from chicken muscle supported by ≥ 2 solid junction reads**

| **Chr** | **Location of**  **5' donor site** | **Location of**  **3' acceptor site** | **No. of forward splice junction reads** |
| --- | --- | --- | --- |
| Chr1 | 59,010,299 | 59,014,491 | 153 |
| Chr15 | 3,336,752 | 3,341,860 | 140 |
| Chr1 | 13,428,346 | 13,432,329 | 60 |
| Chr10 | 19,134,521 | 19,139,879 | 51 |
| Chr15 | 3,336,752 | 3,347,646 | 18 |
| Chr1 | 6,004,457 | 6,024,602 | 16 |
| Chr12 | 5,169,161 | 5,171,633 | 15 |
| Chr1 | 49,693,065 | 49,699,093 | 15 |
| Chr1 | 192,026,567 | 192,036,651 | 12 |
| Chr1 | 33,536,803 | 33,537,663 | 8 |
| Chr1 | 900,323 | 905,456 | 6 |
| Chr14 | 14,005,314 | 14,011,161 | 5 |
| Chr15 | 3,349,957 | 3,355,090 | 4 |
| Chr10 | 4,611,546 | 4,613,176 | 4 |
| Chr1 | 192,031,057 | 192,038,599 | 4 |
| Chr15 | 3,326,920 | 3,329,024 | 4 |
| Chr15 | 1,114,442 | 1,129,577 | 3 |
| Chr1 | 118,421,374 | 118,424,000 | 2 |
| Chr1 | 70,542,887 | 70,549,029 | 2 |
